# Does use of domestic insecticides undermine public health control strategies?

**DOI:** 10.1016/j.lana.2025.101076

**Published:** 2025-03-29

**Authors:** Walter Fabricio Silva Martins, Lee Rafuse Haines, Martin James Donnelly, David Weetman

**Affiliations:** a Laboratório de Entomologia Médica e Molecular- LEMMol, Universidade Estadual da Paraíba - UEPB, Campina Grande, Brazil; b Department of Vector Biology, Liverpool School of Tropical Medicine - LSTM, Liverpool, UK; c Department of Biological Sciences, University of Notre Dame - ND, Indiana, USA

**Keywords:** Household insecticides, Insecticide resistance, Vector-borne diseases, Vector control

## Abstract

Vector-borne diseases (VBD), particularly dengue and malaria, pose a growing threat to human health worldwide. While insecticides remain the cornerstone of vector control programmes, their efficacy is being compromised by increasing insecticide resistance in mosquito populations, leading to control failures that have significant epidemiological and socioeconomic implications. Current research has predominantly examined resistance development in the context of public health interventions and agricultural applications. However, the contribution of domestic insecticide use to resistance evolution in VBD-endemic regions remains inadequately characterised. Evidence indicates that household insecticide utilisation is extensive, with approximately 60% of residents in endemic areas regularly employing domestic insecticidal products for personal protection. This viewpoint highlights how the poorly regulated household insecticide market may significantly contribute to resistance development. Therefore, understanding the impact of domestic insecticide products and usage patterns is urgently needed to preserve the efficacy of vector control campaigns and protect public health outcomes.

## Introduction

Mosquitoes of the genera *Anopheles*, *Aedes*, and *Culex* are responsible for transmitting diseases to which 80% of the global population is at risk.^1^^,^^2^ Multiple factors have contributed to the sustained transmission of vector-borne diseases (VBD) worldwide, such as the mosquitoes' ability to adapt to urban environments, poor water security leading to mosquito-friendly water storage, and extreme weather caused by climate change (e.g., rainfall and droughts). Human migration, immunological susceptibility, limited financial resources, and the lowered effectiveness of preventive approaches also play a role in disease transmission. Projections suggest that rising temperatures could put more than eight billion people at risk of dengue and malaria within the next 60 years, with transmission in some areas moving from seasonal to year-round.^3^

Despite significant global investment, VBD transmission remains high, and the incidence of dengue has increased by 30-fold worldwide over the past 50 years.^4^ Additionally, since 2014, *Aedes*-transmitted viral diseases, such as chikungunya and Zika, have rapidly emerged or re-emerged. For instance, chikungunya autochthonous transmission was reported in Europe (France and Italy) in 2017, with dengue outbreaks in France in 2014, 2015 and 2023.^5^, ^6^, ^7^ Another serious concern is the invasion of the Asian mosquito, *Anopheles stephensi,* into Africa where it has been linked to urban malaria outbreaks.^8^

Public health strategies to curb VBDs are limited by the restricted availability of effective vaccines, drugs and antiviral therapies and the evolution of behavioural and insecticide resistance. The spread of insecticide resistance in mosquitoes is driven by mechanisms that help the insects survive or avoid exposure to standard insecticide doses that were once lethal, thereby compromising previously efficacious control (detailed in Panel 1). In particular, mosquito resistance to pyrethroid insecticides demands heightened attention as pyrethroids remain a crucial insecticide class for indoor control interventions, such as insecticide-treated bed nets (ITNs).^9^

Panel 1 **Mechanisms of insecticide resistance.**

Pyrethroids belong to a class of insecticides targeting the insect voltage-gated sodium channel (VGSC), which causes excessive neuronal stimulation and subsequent death. Pyrethroid resistance is associated with the development of three main mechanisms that can either act alone or in combination: biochemical (target-site insensitivity and/or metabolic resistance), morphological (cuticular resistance), and behavioural (e.g., avoidance, evasion).

Target-site mutations, known as knockdown resistance (*kdr*), result in structural changes to the VGSC blocking insecticides from binding to the target. In contrast, metabolic resistance involves the over-expression or increased catalytic capacity of metabolic enzymes, predominantly mediated by three detoxification gene families: cytochrome P450s (P450s), esterases, and glutathione S-transferases (GSTs). Biochemical mechanisms are the most extensively studied, but morphological and behavioural resistance can also play an important role by limiting or preventing insecticide absorption, resulting in decreased toxicity. Cuticular resistance is when the mosquito cuticle (exoskeleton) becomes less penetrable to insecticides by thickening and/or altering protein composition. Behavioural resistance produces an enhanced avoidance response in mosquitoes to insecticide-impregnated surfaces. All three mechanisms increase the chances that a mosquito will survive insecticide exposure.

To combat evolving mosquito resistance and reduced effectiveness, health ministries have reduced the use of pyrethroids by 50% over the past decade across intervention campaigns.^9^ This reduction has been achieved by using alternative insecticide classes for indoor residual spraying (IRS).^9^ While this approach should, in theory, decrease pyrethroid resistance in mosquito populations, recent studies with Brazilian *Aedes aegypti*, Ghanaian *Anopheles funestus* and *Anopheles gambiae* have shown the opposite, with escalating pyrethroid resistance.^10^^,^^11^ Although it may be possible that this persistent resistance is driven by the pyrethroids used in public health campaigns such as IRS and ITNs, the poorly regulated use of pyrethroid-based household products such as coils, electric emanators, and aerosols may represent a largely unappreciated contributing factor.

It is challenging to identify the specific factors contributing to the development and spread of pyrethroid resistance, given the various sources of selection pressure, including public health initiatives, private sector practices, and domestic use for personal protection. Studies on domestic insecticides' impact on evolving resistance have been scarce. Therefore, it is crucial to investigate the potential risks associated with the widespread use of household insecticide sprays as it is plausible they contribute to resistance evolution, much like the often-unrestricted use of antibiotics has led to antimicrobial resistance. Indeed, a recent WHO (World Health Organization) guideline for domestic insecticide management highlights the risks of unregulated use of household insecticide in the evolution of insecticide resistance in mosquitoes.^12^

**Fig. 1:**
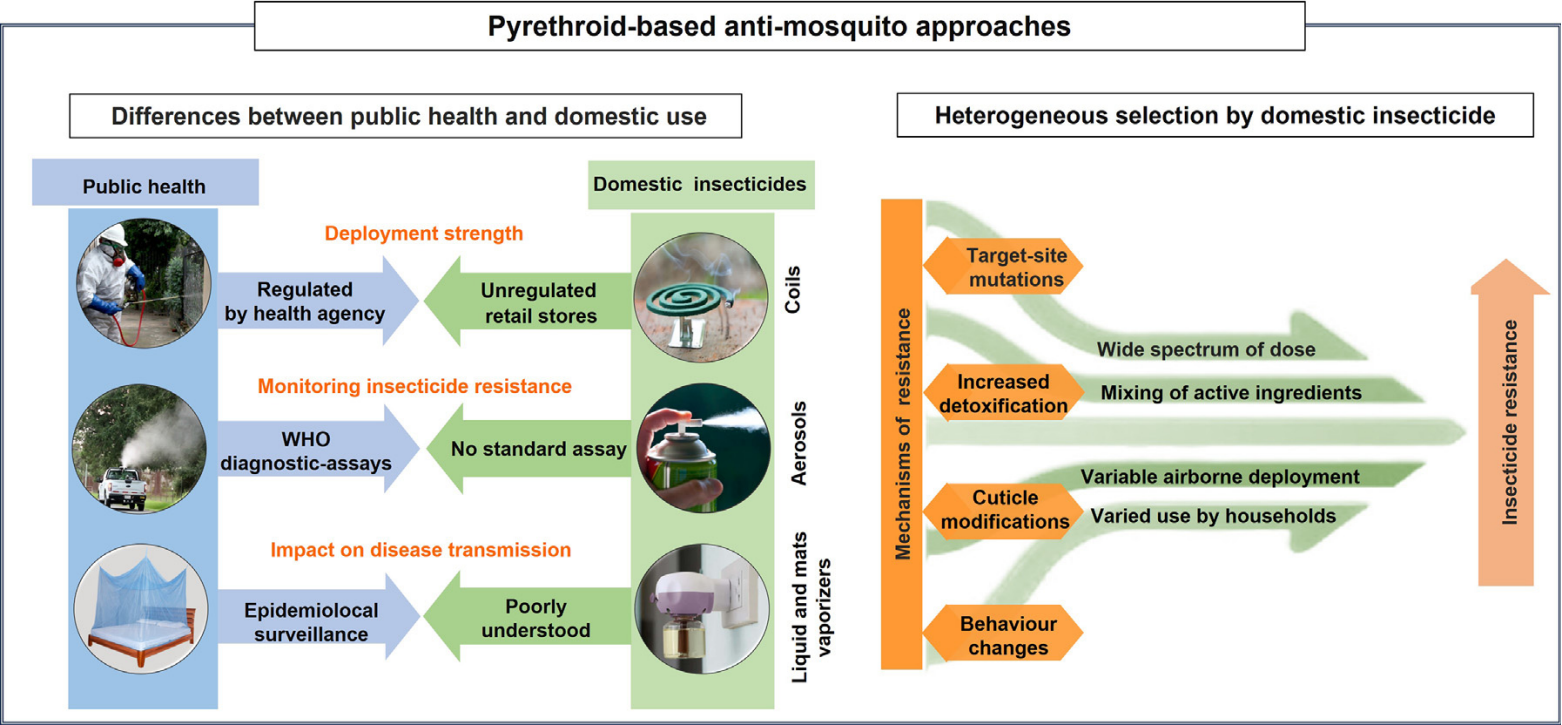
**Drivers of pyrethroid resistance in vector-borne mosquitoes**. Schematic representation of the differences between public health programmes and domestic insecticide usage (left side), and the drivers of evolving mosquito resistance to pyrethroids (right side).

In this viewpoint, we present evidence that household insecticides may contribute to evolving insecticide resistance in mosquito vectors. We also provide an overview of published studies that reveal the widespread use of pyrethroid-based domestic insecticides for self-protection and discuss the characteristics of household insecticides (Fig. 1) that may foster the evolution of resistance.

## Impact of household insecticides on mosquitoes evolving resistance to pyrethroid

**Fig. 2:**
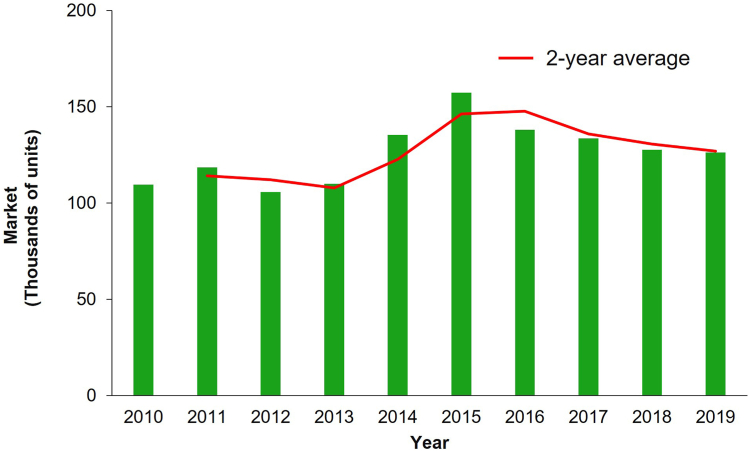
**Estimated country-level use of domestic insecticides in Brazil from 2010 to 2019.** This figure was created based on ABIPLA (Associação Brasileira das Indústrias de Produtos de Higiene, Limpeza e Saneante) annual reports available at http://abipla.org.br/anuario.

**Fig. 3:**
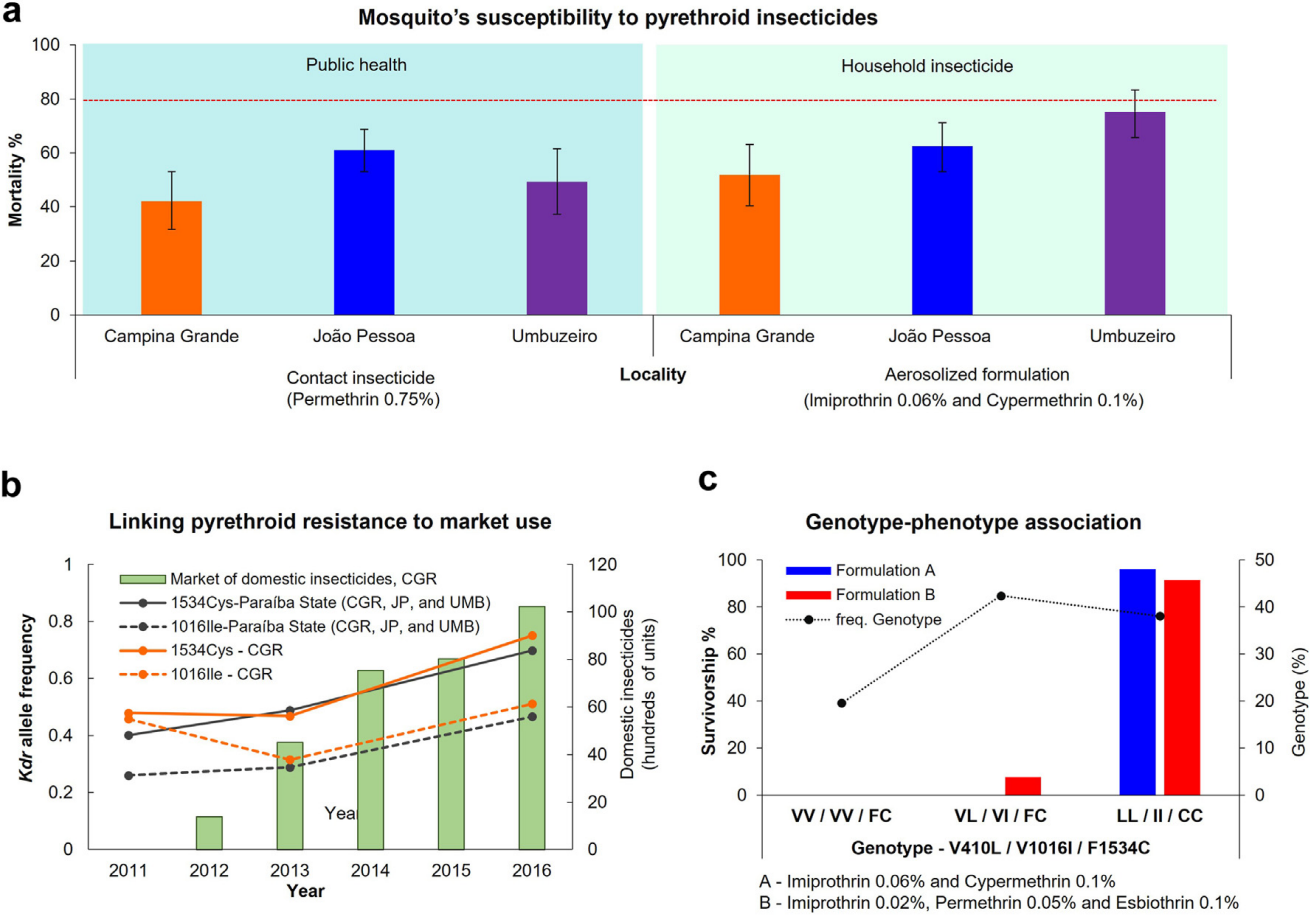
**A case study of resistance to domestic insecticides in Brazilian *Ae. aegypti***. (a) Susceptibility profiling of *Ae. aegypti* from Paraíba State, Northeast Brazil, against pyrethroid insecticides using WHO tube assays (contact insecticide) and Peet Grady chamber assays (aerosolized formulation). The red dashed line represents the WHO threshold for effectiveness at 80% mortality. (b) Association between the growing domestic insecticide market over five years and frequency of *kdr*-resistant alleles in *Ae. aegypti* from Paraíba State. (c) Association between the *kdr*-resistant alleles and whether they reflect resistant phenotypes in *Ae. aegypti*. The case study research approach is outlined in Panel 2.

In Brazil, current nationwide government-led interventions focus on treating larval habitats with pyriproxyfen, a juvenile hormone analogue, and focal fogging with the organophosphate malathion at transmission hotspots.^11^ However, between 2010 and 2015, there was up to a 50% increase in sales of domestic pyrethroid-insecticidal products in the country (Fig. 2). The selected example of *Ae. aegypti* in Paraíba State, Brazil, provides genetic and phenotypic evidence of an apparent link between domestic insecticide use and selection of pyrethroid resistance (Fig. 3a–c). This example provides evidence that domestic insecticide use is driving the selection of resistance in mosquito populations, supported by: *i*) there is no public health use of pyrethroid insecticides (or insecticides that may confer cross-resistance) and no widespread agricultural use in the highly urbanised study locations, *ii*) mosquitoes from three municipalities were resistant to aerosolized pyrethroid-based domestic insecticide available in Brazil's retailers (Fig. 3a), *iii*) the frequency of pyrethroid-specific resistant markers, *kdr* mutations 1016Ile and 1534Cys, almost doubled within six years following the Zika-fuelled marketing of domestic insecticides in Brazil (Fig. 3b), *iv*) almost 100% of the mosquitoes surviving exposure to domestic insecticides were triple-resistant homozygotes for *kdr*-resistant alleles 410Leu, 1016Ile and 1534Cys (Fig. 3c), which enhances survival of *Ae. aegypti* to non-volatile contact pyrethroids.^13^^,^^14^ To our knowledge, this strong associative evidence occurred without other sources of pyrethroid selection.

Unsurprisingly, with the sustained use of domestic insecticides in Brazil (Fig. 2), a similar pattern of evolving pyrethroid resistance in *Ae. aegypti* mosquitoes has also been observed throughout the country, with an overall increase of 27.8% in *kdr* allele frequency (1016Ile + 1534Cys) between 2009 and 2012 and 2017/2018.^11^ Haddi and colleagues, 2017,^15^ also reported a high nationwide frequency (83.1%) of *kdr*-resistant alleles and a high frequency of a recently identified Val410Leu mutation associated with pyrethroid resistance.^15^ From 2004 to 2014, an increase in the frequency of *kdr*-resistant alleles 1016Ile and 1534Cys was also observed in *Ae. aegypti* from various localities in São Paulo State, Brazil.^16^

In support of a link between domestic insecticide use and resistance evolution, semi-field studies have also documented an association of *kdr* alleles with resistance to household aerosolized insecticides. For instance, in *Ae. aegypti* from Thailand, 1016Gly and 1534Cys *kdr* mutations were linked to resistance against aerosol formulations containing blends of pyrethroids.^17^ In Mexico, the 1016Ile mutation was associated with *Ae. aegypti* resistance to two household aerosolized pyrethroid-based formulations.^18^ In Thailand, the mutation 1014Phe in *Culex quinquefasciatus* was linked to resistance against four aerosolized products with pyrethroid blends.^17^ Studies addressing the strength of domestic insecticide selection pressure on *kdr*-resistant alleles are scarce, which may reflect technical requirements for testing aerosolized and volatile formulations that restrict testing capacity, such as the need for controlled environments.^19^^,^^20^

Collectively, these findings suggest that household insecticide usage can contribute to the development of pyrethroid resistance in mosquitoes. They also prompt the question of whether unregulated usage of domestic insecticides in areas with a high burden of VBDs could lead to the selection of cross-resistance to the pyrethroids that are essential for public health anti-mosquito programmes.

### Invisible and indiscriminate use of pyrethroids

Due to the environmental and health risks of insecticide overuse and evolving insecticide resistance, governments and health authorities recommend strict regulations.^21^ However, pyrethroid-based domestic insecticides, accepted as safe for indoor use, lack post-market effectiveness oversight.

**Fig. 4:**
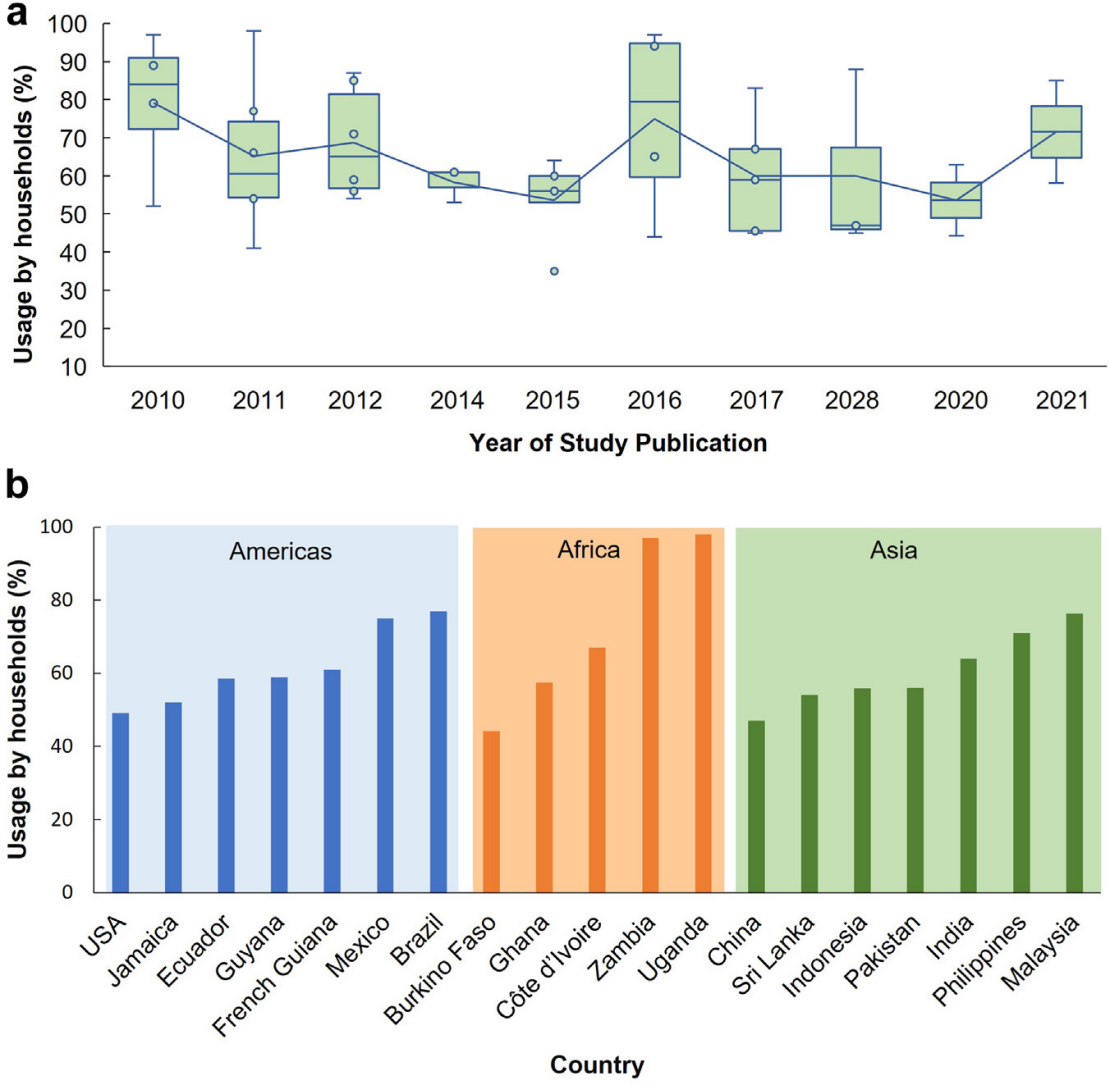
**Proxies for domestic insecticide use for self-protection in vector-borne diseases endemic countries**. (a) Average usage of domestic insecticides across 19 countries over a decade. (b) Country average use of household insecticides from 2010 to 2021. These figures were created based on a literature review, and citations for studies included are provided in Supplementary Table S1.

The WHO has tracked the global use of insecticides for public health, which revealed a two-fold decrease in global pyrethroid use from a peak during the last decade.^9^ However, specific data on the local use of household insecticides is scarce, and it remains unclear who manages such data. Data compiled on domestic insecticide use (detailed in Panel 2) indicate that household insecticides have been commonly used for self-protection over the past decade in the 19 countries for which data are available. Across the Americas, Africa and Asia, approximately 60% of the homeowners surveyed used one or more insecticide-based products (e.g., aerosols, coils, and spatial repellents) (Fig. 4a and b). How vector-borne diseases influence homeowner attitudes in terms of self-protection can be illustrated by the impact of the 2014–2015 chikungunya and Zika outbreaks in Brazil. There was a 50% increase in domestic insecticide sales in the Brazilian market during the outbreaks (Fig. 2). As both diseases were associated with severe illnesses, including Guillain-Barré syndrome in adults and microcephaly in newborns, people responded by protecting themselves from the diseases through mosquito elimination measures.

Panel 2 **Data search strategy.**

To understand the publication landscape addressing the use of household (domestic) insecticides for mosquito control, we conducted a literature review by searching Google Scholar. Our search terms included “household insecticides” or “domestic insecticides” AND “arbovirus” or “vector-borne diseases” AND “attitude and practice” published between 2010 and 2023. We then screened the returned articles for inclusion criteria such as personal protection usage, active ingredients, and bioefficacy against vector mosquitoes. In total, we retrieved 53 articles from 29 countries, of which 15 described the product formulations and seven studies assessed mosquito susceptibility to domestic insecticides.

For the pyrethroid resistance case study in Brazil, we obtained city-level (Campina Grande–CGR, Paraíba State) local retail shops’ annual sales records for aerosols, electric vaporisers, liquid, fumigation gas, and coils. We then merged all data to estimate the use of pyrethroid via domestic insecticides between 2011 and 2016, corresponding to pre- and post-Zika and Chikungunya outbreaks in Brazil.

To determine mosquito susceptibility to commercial household insecticides, we conducted insecticide exposure assays and recorded insect mortality rates of three disease-spreading mosquito species. In total, we tested four pyrethroid-resistant populations, three laboratory colonies, and one Brazilian *Ae. aegypti* field population from CGR. Mosquitoes were assessed against two commercial household pyrethroid-based aerosolized insecticides as described by Silva Martins et al., 2023.^20^ Alive and dead mosquitoes were tested for three pyrethroid-resistance-associated target-site mutations (Val410Leu, Val1016Ile, and Phe1534Cys) in the voltage-gated sodium channel gene (*Vgsc*) to assess their association with mosquito resistance to aerosolized insecticides. Each mosquito was genotyped at these loci using the TaqMan assays (Life Technologies, UK). The mosquito colonies were maintained and provided by LITE (Liverpool Insect Testing Establishment), and information on their susceptibility to non-volatile contact pyrethroids is provided at https://lite.lstmed.ac.uk/mosquito-colonies.

This consumer behaviour was not restricted to Brazil; 45% of households in Texas, USA, reported using insecticidal products when elevated disease risks were perceived.^22^ In countries where one or multiple insect-borne diseases are endemic, such as Brazil, Mexico, Zambia, Uganda and Malaysia, homeowners' use of domestic insecticides has reached levels as high as 74–94% over the last decade (Fig. 4b).^18^^,^^23^, ^24^, ^25^ While herein we focus on the impact of recent *Aedes*-transmitted arbovirus outbreaks on the growing market of domestic insecticides, the adoption of insecticide products for self-protection has been a long-term practice in VBD-endemic countries, which happened alongside significant historical public health challenges to curb persistent disease outbreaks.^1^

The widespread household adoption of domestic insecticides, depicted in Fig. 4a and b, may be motivated by controlling nuisance-biting mosquitoes and disease prevention. In effect, this human behaviour of homeowners self-protecting their homes also reflects the reality that public health interventions are not effectively controlling mosquito populations. Reasons for this are complex and involve various factors, including inadequate control coverage, ineffective interventions, and sustained insecticide resistance. For instance, attempts to control urban mosquito populations, like *Ae. aegypti* and *Ae. albopictus,* by targeting larval habitats with larvicides, are often compromised by homeowners who store water in artificial standing water containers. These practices could help fuel homeowners' use of domestic insecticides to control mosquito populations in and around their homes.

Meanwhile, despite continuous anti-mosquito campaigns with IRS and ITNs in several African countries, domestic insecticides are still heavily used, even in combination with ITNs, as reported in Côte d’Ivoire and Zambia.^26^^,^^27^ Use of domestic insecticides was linked to the lack of good ITN coverage in households, concerns about disease transmission and mosquito nuisance.^26^^,^^27^

Given the wide range of insecticide-based domestic products (e.g., coils, electric emanators, and aerosols) formulated to target a broad range of insects, tracking the composition and efficacy of each formulation against native mosquitoes is another essential piece of the puzzle to ensure effective risk management of domestic insecticides.

### A plethora of heterogeneous-insecticidal selection

Untangling the drivers of evolving insecticide resistance remains a complex task as interactions across biological and environmental factors and the strength of insecticidal selection must be considered.^28^ The unregulated usage of domestic insecticides adds further complexity to this already dynamic process, as selection strength will vary at a fine scale within households and among neighbours, depending on insecticide use behaviours and formulation compositions, as highlighted in Fig. 1.

**Fig. 5:**
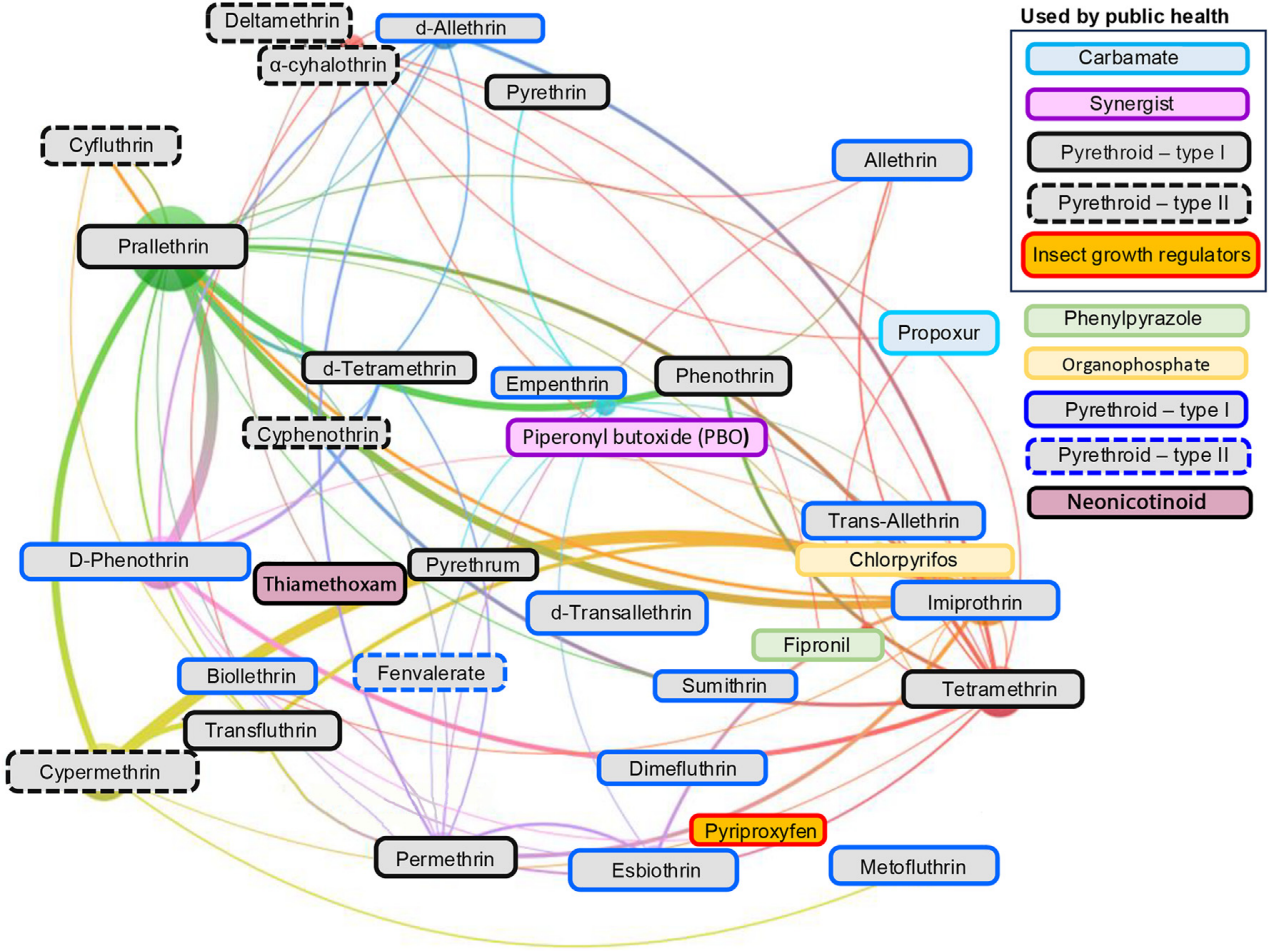
**Mosquitocidal active ingredients in aerosolized domestic insecticides.** Edge weight represent the frequency and co-occurrence of compounds across formulations, respectively. This figure was created based on the chemical composition described by product manufacturers and published studies as detailed in Supplementary Table S3.

The vast spectrum of aerosolized insecticide formulations identified globally (Fig. 5) is a major concern due to the potential for heterogeneous selection pressure and evolution of cross-resistance. Across 326 worldwide insecticidal products containing a blend of two or three active ingredients, we identified 67 distinct formulations. A network analysis based on the co-occurrence of insecticidal active ingredients in aerosolized domestic products highlights the complex interactions between compounds, posing challenges for managing insecticide resistance (Fig. 5). Notably, pyrethroids, widely used in public health, are also prevalent in domestic formulations, increasing the risk of co-evolution of resistance that could undermine official vector control intervention.

The predominant blends consisted of different pyrethroid sub-classes (type I and II), with occasional use of other insecticide classes and synergist compounds. This creates a favourable landscape for selecting pre-existing and alternative insecticide-resistance-associated mechanisms.^29^^,^^30^ Within the mix of domestic insecticide formulations, metabolic resistance can broaden the evolution of cross-resistance due to the vast repertoire of detoxification genes like P450 gene families, which are capable of metabolising pyrethroids (type I and II), carbamates and pyriproxyfen, an insect growth regulator.^31^, ^32^, ^33^

Indeed, mosquitoes’ susceptibility varies among populations (Fig. 3a) and among co-endemic vector species, ranging from resistant *Ae. aegypti* and *An. gambiae* to susceptible *Cx. quinquefasciatus* (Supplementary Figure S1). Also, semi-field testing has reported reduced effectiveness of domestic insecticide against *Ae. aegypti* and *Ae. albopictus* from a distinct geographic/genetic background (Supplementary Figure S2, Supplementary Table S2). Furthermore, susceptibility testing in *Ae. aegypti,* from Morales, Mexico, against 13 household aerosol insecticides revealed that formulations' effectiveness (30–100% mortality) can reflect the blending and concentration of pyrethroids.^34^

However, it is also important to consider that inappropriate household use, such as deploying lower doses than recommended, could also lead to heterogeneous selection pressure by oscillation between optimal and suboptimal doses. For instance, as shown in Supplementary Figure S1 and elsewhere,^12^^,^^35^ mosquito colony susceptibility reflects a dose–response of aerosolized formulation discharge. The compromised efficiency of household insecticides could also strengthen selection pressure; households may apply or reapply products too frequently, use higher doses than recommended, or combine different insecticidal products.

### Domestic pesticide management—how urgent is the need?

Before any recommendation for potential restrictions on domestic pesticide use is considered, knowing the potential impact of self-protection in preventing the transmission of vector-borne diseases in local communities is essential. For example, despite evolving *kdr* resistance in mosquitoes, volatile pyrethroids in domestic insecticides (e.g., coils, vaporisers, and aerosols) could still provide adequate spatial repellent protection.^36^ In the meantime, promoting non-chemical control and rational use of insecticides through public educational campaigns is a feasible starting point to preserve the efficacy of public health interventions and minimise potential adverse environmental effects. For instance, high levels of atmospheric air pollution by pyrethroids have been recently identified in Brazilian urban and semi-urban settlements.^37^^,^^38^

From a classical public health resistance management perspective, the likely impact of unregulated domestic insecticide use has been considered minimal compared to using insecticides for vector control and agriculture. However, recent field evidence has highlighted a neglected risk, described by the Brazilian vector control programme. The overuse of domestic insecticides in Rio de Janeiro, Brazil was linked to the failure of a promising non-insecticidal intervention using *Wolbachia*-infected *Ae. aeygpti.*^18^^,^^39^ This suggests that the overall in-country efficacy of intervention programmes can be jeopardised, wasting already limited financial and human resources available for vector control.

With increasing usage of domestic pyrethroid-insecticidal products for personal protection, further reports of evolving resistance and its impact on vector control are expected. Our findings highlight the urgent need for standardized methods to test mosquito populations against domestic insecticides. However, assessing volatile or aerosolized products remains challenging, often requiring controlled environments like the Peet Grady chamber (PG-chamber).^19^ A higher-throughput methodology for testing aerosolized products was recently developed by Silva Martins et al., 2023,^20^ but meaningful benchtop bioassays are crucial for improving resistance management and decision-making.^40^ Given the limited understanding of pyrethroid-based domestic insecticides' impact on resistance, the future role of pyrethroids in vector control remains uncertain without better management.

## Conclusions

As global public health faces the challenge of VBDs and increasing difficulties in mitigating transmission due to evolving insecticide resistance, the widespread use of domestic insecticides described in this viewpoint highlights the urgent need to investigate their direct and indirect impacts on public health campaigns. We advocate for further research on the extent and effects of domestic insecticide use to elucidate potential environmental impact, risks and benefits to human health, and implications for vector control programmes. Based on existing evidence, we recommend the WHO develop further guidance for household pesticide management and assist in-country policymakers in improving legislation throughout the product life cycle. Also, industry engagement through post-market monitoring would ensure products are optimally deployed to mitigate resistance. Additionally, enhancing laboratory facilities to test mosquito susceptibility to insecticides is crucial to verify product effectiveness against native mosquitoes and monitor evolving resistance.

Finally, despite challenges in managing domestic pesticide production and use, collaboration among public health authorities, stakeholders, industry, and academics is essential for a global initiative that supports evidence-based decisions prioritizing environmental safety, household well-being, and sustainable vector control campaigns.

## Contributors

WFSM conceptualised this viewpoint, wrote the original draft, performed the study design, data collection and curation, analysis and visualisation of the data, and funding acquisition. LRH contributed to data analysis and figure visualisation and reviewed and edited the manuscript. MJD and DW reviewed and edited the manuscript. All authors read, revised, and approved the manuscript.

## Declaration of interests

The authors declare that they have no competing interests.

## Appendix A Supplementary data

Translated Summary Portuguese

Supplemetary Figures and Tables
