# Supplementary material for: Does use of domestic insecticides undermine public health control strategies?
Source: Lancet Reg Health Am. 2025 Mar 29;45:101076. doi: 10.1016/j.lana.2025.101076 (PMC11994388; doi:10.1016/j.lana.2025.101076)
Supplement: Translated Summary Portuguese [file mmc1.pdf]

__Editorial Disclaimer:__ This translation in Portuguese was submitted by the authors and we reproduce it as supplied. It has not been peer reviewed. Our editorial processes have only been applied to the original abstract in English, which should serve as a reference for this manuscript

Resumo

As doenças transmitidas por mosquitos vetores (VBD), como dengue e malária, representam uma ameaça crescente à saúde pública global. O controle desses mosquitos depende amplamente do uso de inseticidas, embora a crescente resistência dos mosquitos a essas substâncias tenha levado à ineficácia das intervenções de controle, resultando em impactos significativos na saúde humana e no desenvolvimento econômico. Embora a maioria dos estudos enfoque a resistência induzida pelo uso de inseticidas na saúde pública e na agricultura, o papel dos inseticidas domésticos em regiões endêmicas permanece amplamente negligenciado. Este manuscrito apresenta evidências de que o mercado crescente e pouco regulamentado de inseticidas domésticos pode contribuir significativamente para a resistência dos mosquitos transmissores de doenças. Cerca de 60% dos domicílios em áreas endêmicas utilizam regularmente inseticidas domésticos, criando uma intensa pressão de seleção. Ao analisar as características desses produtos e seus padrões de uso, ressaltamos a urgência de avaliar o impacto dessa pressão de seleção para reduzir os efeitos negativos nas campanhas de controle vetorial e na saúde pública.
