# Supplementary material for: Does use of domestic insecticides undermine public health control strategies?
Source: Lancet Reg Health Am. 2025 Mar 29;45:101076. doi: 10.1016/j.lana.2025.101076 (PMC11994388; doi:10.1016/j.lana.2025.101076)
Supplement: Supplemetary Figures and Tables [file mmc2.pdf]

# Does use of domestic insecticides undermine public health control strategies?

Walter Fabricio Silva Martins[1,2], Lee Rafuse Haines[2,3], Martin James Donnelly[2], David Weetman[2]

[1]Laboratório de Entomologia Médica e Molecular- LEMMol, Universidade Estadual da Paraíba - UEPB, Campina Grande, Brasil. [2]Department of Vector Biology, Liverpool School of Tropical Medicine - LSTM, Liverpool, UK. [3]Department of Biological Sciences, University of Notre Dame - ND, Indiana, USA.

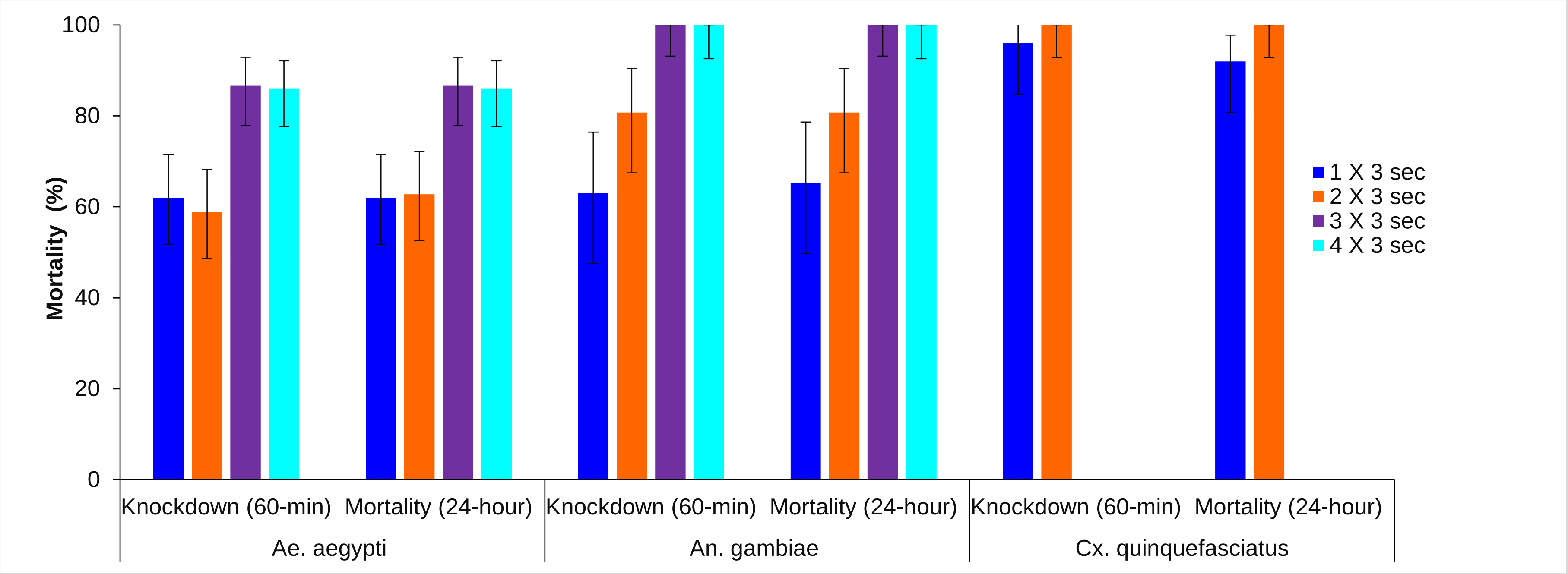


**Figure S1: Susceptibility profiling of three mosquito colonies against domestic insecticide. The red dashed line represents the WHO threshold for effectiveness at 80% mortality.** All resistant colonies: *Aedes aegypti* (Cayman), *Anopheles gambiae* (Tiassale) and *Culex quinquefasciatus* (Muheza) were maintained and provided by LITE (Liverpool Insect Testing Establishment). Colonies' susceptibility to non-volatile contact pyrethroids is provided at https://lite.lstmed.ac.uk/mosquito-colonies.

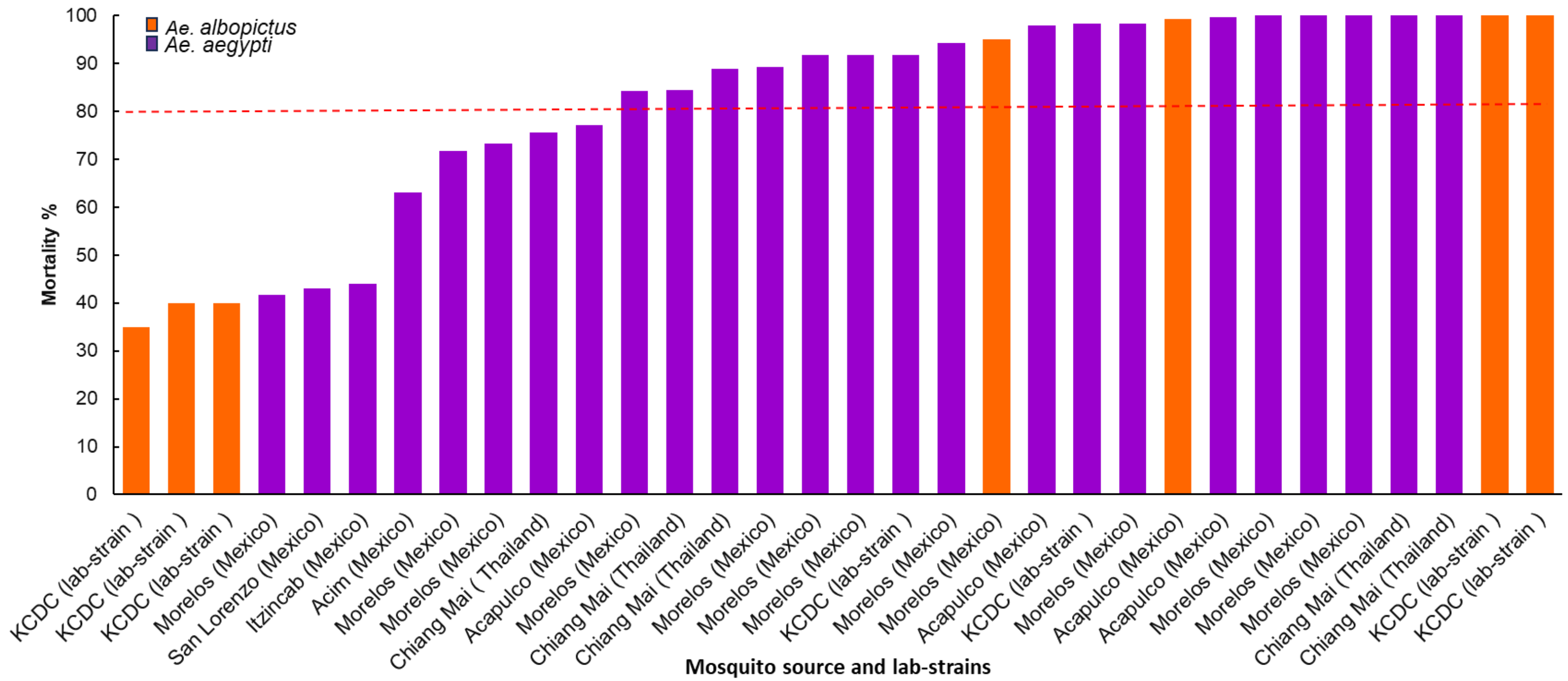


**Figure S2: Susceptibility of *Ae. aegypti* mosquitoes exposed to aerosolized domestic insecticides in semi-field conditions.** The red dashed line represents the WHO threshold for effectiveness at 80% mortality. Orange bars represent *Aedes albopictus*, and purple represents *Aedes aegypti*. This figure was created using the data extracted from a literature review (Supplementary Table S3).

**Table S1**. Household insecticide use in countries with endemic vector-borne diseases

| Country | Year | Region | Locality | Overall domestic insecticide usage | Aerosol | Electric vaporizer/ repellent | Coil | Reference |
|---|---|---|---|---|---|---|---|---|
| Australia | 2014 | Western | Perth, Gascoyne, Goldfields-Esperance, Great Southern, Kimberley, Midwest, Pilbara, Southwest, and Wheatbelt | 58.85% | 33.2% | 93.30% | 58.85 | [1] |
| Brazil | 2010 | Rio de Janeiro | Niterói | 79% | 66.96% | | | [2] |
| | 2012 | Piauí | Picos | 85% | 70.7% | | 1% | [3] |
| | 2015 | Rio de Janeiro | Rio de Janeiro metropolitan region | 87.50% | 38.10% | | | [4] |
| | 2020 | Roraima | Boa Vista | 62.88% | | | | [5] |
| | 2022 | Nationwide | Cross-sectional | 70.38% | 32.00% | | | |
| China | 2018 | Hong Kong | Cross-sectional | 47.20% | 18.80% | 19.80% | 8.60% | [6] |
| Côte d'Ivoire | 2017 | Southern | Elibou | 67% | 6.4% | | 15.30% | [7] |
| Ecuador | 2011 | El Oro | Machala | 77% | | 51% | | [8] |
| | 2015 | El Oro | Machala | 53% | | 32% | | [9] |
| | 2017 | Southern | Machala | 27.50% | | | | [10] |
| | | | Huaquillas | 18.90% | | | | |
| | | | Portovelo | 45.60% | | | | |
| | | | Zaruma | 36.50% | | | | |
| French Guiana | 2014 | Sain Lauren, Mana, Kourou, Matorou, Cauenne. | Cross-sectional | 60.50% | 34.70% | | | [11] |
| Ghana | 2011 | Southern Ghana | Cross-sectional | 54.75% | 26.00% | | 63% | [12] |
| | 2014-2015 | Southern Ghana | Shai-Osudoku district | 59.80% | | | | [13] |
| Greece | 2016 | cross-sectional | cross-sectional | 46.20% | | 46.2% | | [14] |
| Guyana | 2017 | cross-sectional | cross-sectional | 59.31% | | 39.83% | 19.48% | [15] |
| India | 2010 | Tamil Nadu | Nemam village | 96.50% | | | 75.0% | [16] |
| | 2011 | Mumbai | cross-sectional | 66.0% | | | | [17] |
| | 2011 | Rajkot district | cross-sectional | 52.30% | | | 13.4% | [18] |
| | 2014 | Saurashtra | Bhavnagar | 52.50% | | | 52.2% | [19] |

| | | | | | | | | |
|---|---|---|---|---|---|---|---|---|
| | 2016 | Karnataka | Manjanady, Asaigoli, and Kuthar villages | 65.0% | 4.5% | | 65.0% | [20] |
| | 2017 | Telangana | Hyderabad | 45% | 1.5% | | 45.0% | [21] |
| | 2021 | Kerala | Ponekkara | 58.1% | 58.1% | | | [22] |
| | 2008/2010 | Jharkhand and Bihar | cross-sectional | 73.2% | | | 12.4% | [23] |
| | 2010-2011 | Maharashtra | cross-sectional | 73.0% | | 28.0% | 33.0% | [17] |
| | 2012-2015 | Tamil Nadu, Kheda and Sundargarh | Chennai, Nadiad and Raurkela | 64.3% | | 44.2% | 41.9% | [24] |
| Indonesia | 2012 | Central Java Province | Semarang, Kudus, Jepara and Surakarta | 56% | | | | [25] |
| | 2012 | Ambarawa | kupang village, Tambakboyo village, and Panjang village | 58% | | | | [26] |
| | 2015 | Yogyakarta | Sleman | 35,4% | 34% | 34,7% | 31,3% | [27] |
| | 2016 | Yogyakarta | Bantul | 43,7% | 31.3% | 39,6% | 29,4% | |
| | 2017 | Yogyakarta | Gunung Kidul | 44,7% | 10.9% | 37,5% | 51,6% | |
| | 2017 | | Jawa Tengah | 83.30% | 10% | 30% | | [28] |
| | 2014-2015 | Yogyakarta | Mantrijeron and Demangan | 61% | | 21.60% | | [29] |
| Jamaica | 2010 | Western Region | St. James, Westmoreland, Hanover, and Trelawny | 52% | | | 52% | [30] |
| Malaysia | 2010 | Selangor and Kuala Lumpu | Sungai Buloh, Rawang, Batu, Setapak, and Hulu Kelang. | 88.70% | | 88.70% | 59.00% | [31] |
| | 2011 | Negeri Sembilan | Seremba | 54% | 54.16% | | 49.16% | [32] |
| | 2018-2019 | Selangor | Damansara Damai | 95% | 62% | | | [33] |
| | 2021 | Selangor | cross-sectional | 85.00% | 85% | 50.10% | 74.30% | [34] |
| | 2021 | Selangor | cross-sectional | 74.30% | | 50.10% | 68% | |
| | 2012-213 | Cross-sectional | cross-sectional | 59% | | | | [35] |
| Mexico | 2012 | Guerrero | cross-sectional | 44.20% | | | | [36] |
| | 2012 | Yucatán | Mérida | 86.60% | 73.60% | 37.40% | 28.30% | [37] |
| | 2016 | Yucatán | Mérida | 94% | 86.50% | 6.40% | 6.40% | [38] |
| Pakistan | 2015 | Peshawa | Khyber Pakhtunkhwa | 56.34% | 24.67% | 31.67% | | [39] |
| Philippines | 2012 | Samar | cross-sectional | 70.90% | 31.58% | | 70.90% | [40] |
| Sri Lanka | 2017 | Northern Province | Jaffna | 54.00% | 15.50% | 4.50% | 34% | [41] |
| Uganda | 2011 | Kira | cross-sectional | 98% | 71.40% | | 38.80% | [42] |
| USA | 2012 | Florida | Key West | 40.80% | 40.80% | | 14.80% | [43] |
| | 2012 | Arizona | Tucson | 30.10% | 30.10% | | 6.70% | [43] |

| | | | | | | | | |
|---|---|---|---|---|---|---|---|---|
| | 2018 | Texas | San Antonio | 45% | | 45% | | [44] |
| Zambia | 2016 | Nyimba and Luangwa | Cross-sectional | 97% | 97% | | | [45] |

**Table S2.** Chemical composition of household aerosol insecticides used in different countries

| Year | Country | Product | Product Description | Insecticide | Natural extracts/synergist | Reference |
|---|---|---|---|---|---|---|
| 2023 | Argentina | RAID | Raid Kills Flies, Mosquitoes and Mosquitoes | cypermethrin 0.1%, pralethrin 0.1%, transfluthrin 0.1% | | This study |
| 2023 | | | Raid Essentials Home & Garden | pyrethrin 0.15%, piperonyl butoxide (PBO) 0.63% | PBO | This study |
| 2023 | | | Raid House & Garden | d-phenothrin 0.131%, pralethrin 0.109% | | This study |
| 2023 | Australia | RAID | Raid® Max Multi Insect Killer with Double Nozzle Technology | 0.50 g/kg imiprothrin, 0.50g/kg prallethrin, 0.15g/kg cyfluthrin | | This study |
| 2023 | | | Raid® Max Flying Insect Killer with Double Nozzle Technology | 0.50 g/kg imiprothrin, 0.50g/kg prallethrin, 0.15g/kg cyfluthrin | | This study |
| 2023 | Brazil | BAYGON | Inseticida Aerosol Baygon Multi Insetos | prallethrin 0.102%, d-phenothrin 0.125% | | This study |
| 2023 | | | Inseticida Baygon Ação Total Aerosol | prallethrin 0.03%, cypermethrin 0.1%, imiprothrin 0.03% | | This study |
| 2023 | | | Inseticida Mata Baratas Baygon | imiprothrin 0.06%, cypermethrin 0.10% | | This study |
| 2023 | | | Inseticida Aerossol Ultra Embalagem Econômica Baygon | prallethrin 0.03%, cypermethrin 0.1%. imiprothrin 0.03% | | This study |
| 2023 | | | Inseticida Aerosol Ação Total Óleo De Eucalipto Baygon | prallethrin 0.03%, cypermethrin 0.1%, imiprothrin 0.03% | eucalyptus oil | This study |
| 2023 | | DETEFON | Inseticida Aerosol Detefon Ação Total | imiprothrin 0.015%, permethrin 0.069% | | This study |
| 2023 | | FERRACUPIM | Inseticida Imunizante Ferracupim Nobel | fenvalerat 0.35 % | | This study |
| 2023 | | JIMO | Jimo Open Air Spray 300 Ml | transfluthrin | | This study |
| 2023 | | | Inseticida Jimo Ácaros E Pulgas Spray | d-phenothrin, pyriproxyfen | | This study |
| 2023 | | | Inseticida Jimo Anti-Inset Multiinseticida | cypermethrin 0.1% | | This study |
| 2023 | | | Inseticida JIMO Cupim Aerosol | cypermethrin | | This study |
| 2023 | | | Inseticida JIMO Cupim Incolor Líquido Lata 900ml | cypermethrin | | This study |
| 2023 | | | Inseticida Jimo Anti Traça Aerosol 300ml | empenthrin 0.5% | | This study |
| 2023 | | | Inseticida Da Mata Baratas Aerosol | imiprothrin 0.06%, cypermethrin 0.10% | | This study |

| | | | | | | |
|---|---|---|---|---|---|---|
| 2023 | | KELLDRIN | Inseticida Spray Kelldrin Poderoso | d-allethrin 0.10%, d-tetramethrin 0.10%, d-cyphenothrin 0.13% | | This study |
| 2023 | | MAT | Inseticida Aerosol Mat Inset Mata Barata Ação Total 300ml | cyphenothrin 0.13%, prallethrin 0.06%, imiprothrin 0.04% | | This study |
| 2023 | | | Inseticida Mat Inset Multi Inseticidas Ação Imediata Aerosol | lambda-cyhalothrin 0.5% | | This study |
| 2023 | | | Inseticida Mat Inset Sem Cheiro Aerosol | cyphenothrin 0.12%, metofluthrin 0.03% | | This study |
| 2023 | | | Inseticida Mat Inset Multi Inseticidas | cyphenothrin 0.12%, metofluthrin 0.03% | | This study |
| 2023 | | MORTEIN | Inseticida Aerosol Rodasol Mortein Ação Total | d-phenothrin 0.03%, imiprothrin 0.02%, esbiothrin 0.10% | | This study |
| 2023 | | | Inseticida MORTEIN RODOX Barreira Ativa Aerosol | imiprothrin 0.03%, deltamethrin 0.05% | | This study |
| 2023 | | | Inseticida MORTEIN Mata Baratas Aerosol | imiprothrin 0.07%, cypermethrin 0.19% | | This study |
| 2023 | | | Inseticida Aerosol Ação Total Mortein Pro Frasco Econômica | imiprothrin 0.02%, permethrin 0.05%, esbiothrin 0.10% | | This study |
| 2023 | | | Inseticida Mortein Barreira Ativa Pro Outdoor Aerosol 300ml | imiprothrin 0.03%, deltamethrin 0.05% | | This study |
| 2023 | | | Inseticida Mortein Ação Total Eucalipto Aerosol 400ml | transfluthrin 0.02%, imiprothrin 0.02%, cypermethrin 0.05% | eucalyptus oil | This study |
| 2023 | | | Multi-Inseticida Aerosol Ação Total Pro Mortein 360ml | imiprothrin 0.02%, permethrin 0.05%, esbiothrin 0.10% | | This study |
| 2023 | | PROINSET | Inseticida Proinset Aerosol 400ml | aletrina 0.001%-1%, tetramethrin 0.001%-1%, d-limoneno 0.001%-1% | | This study |
| 2023 | | RAID | Inseticida RAID Ação Total Aerosol | prallethrin, cypermethrin, imiprothrin | | This study |
| 2023 | | | Inseticida RAID Multi Insetos Aerosol | d-allethrin 0.1%, permethrin 0.1%, tetramethrin 0.35% | | This study |
| 2023 | | | Inseticida Raid Protector Mata Barata D-Limoneno 300ml | imiprothrin 0.10%, cypermethrin 0.10% | | This study |
| 2023 | | | Inseticida RAID Protector Mata Baratas e formigas Aerosol | improtrina 0.1%, cypermethrin 0.1% | | This study |
| 2023 | | | Inseticida Multi Insetos Eucalipto Raid 420ml | d-phenothrin 0.12%, d-tetramethrin 0.11%, prallethrin 0.05% | eucalyptus oil | This study |
| 2023 | | | Inseticida Aerosol Multi-Insetos Base Água 420ml | prallethrin 0.102%, d-phenothrin 0.125% | | This study |
| 2023 | | | Inseticida Aerosol Multi-Insetos Aqua Protection | d-phenothrin 0.125%, prallethrin 0.102% | | This study |

| | | | | | | |
|---|---|---|---|---|---|---|
| 2023 | | | Inseticida Aerosol Multi-Insetos Citronela Raid | d-phenothrin 0.125%, prallethrin 0.102% | citronella oil | This study |
| 2023 | | | Inseticida Multi Insetos Raid | prallethrin 0.102%, d-phenothrin 0.125% | | This study |
| 2023 | | | Inset Raid Aero Protection Lv Pg | prallethrin 0.102%, d-phenothrin 0.125% | | This study |
| 2023 | | SBP | Inseticida SBP Casa E Jardim Aerosol Frasco 300ml | d-allethrin 0.297%, d-phenothrin 0.050% | | This study |
| 2023 | | | Inseticida SBP Multi Citronela Aerosol Frasco 300ml | imiprothrin 0.020%, permethrin 0.050%, esbiothrin 0.100% | citronella | This study |
| 2023 | | | Inseticida SBP Multi Inseticida Aerosol Frasco 300ml | d-allethrin 0.135%, d-tetramethrin 0.10%, parmetrina 0.10% | | This study |
| 2023 | | | Inseticida SBP Multi Automático Com 50% De Desconto 250ml | permethrin 0.72%, transfluthrin 0.58% | | This study |
| 2023 | | | Inseticida Multiautomático Óleo Citronela | permethrin 0.8%, transfluthrin 0.6% | citronella | This study |
| 2023 | | | SBP Multiautomático Óleo Eucalipto | permethrin 0.8%, transfluthrin 0.6% | eucalyptus oil | This study |
| 2023 | | | Inseticida Multi-Inseticida Óleo Eucalipto | imiprothrin 0.020%, permethrin 0.050%, esbiothrin 0.100% | eucalyptus oil | This study |
| 2023 | | | Inseticida Multi Insetos Citronela SBP Refil 250ml | permethrin 0.8%, transfluthrin 0.6% | citronella | This study |
| 2023 | | | Inseticida Aerossol Óleo De Citronela SBP 380ml | transfluthrin 0.02%, imiprothrin 0.02%, cypermethrin 0.05% | citronella | This study |
| 2023 | | | Inseticida SBP Anti *Aedes Aegypti* Spray 450ml | transfluthrin 0.040%, permethrin 0.050% | | This study |
| 2023 | | | Inseticida Aerossol À Base De Água Ultra SBP 360ml | transfluthrin 0.02%, imiprothrin 0.02%, cypermethrin 0.05% | | This study |
| 2023 | | | Inseticida Aerossol Sem Cheiro Noites Tranquilas SBP 360ml | transfluthrin 0.02%, imiprothrin 0.02%, cypermethrin 0.05%. | | This study |
| 2023 | | | Inseticida SBP Insetos Base Água 273ml | cepemetrina | | This study |
| 2023 | | | Inseticida SBP Multi Inset Citronela Aerossol 273ml | transfluthrin 0.02%, imiprothrin 0.02%, cypermethrin 0.05% | citronella | This study |
| 2023 | | | Inseticida SBP Multi Suave Aerossol 380ml | transfluthrin 0.02%, imiprothrin 0.02%, cypermethrin 0.05% | | This study |
| 2023 | | | Inseticida SBP Aero Multi Eucalipto Leve 450ml Pague 300ml | transfluthrin 0.02%, imiprothrin 0.02%, cypermethrin 0.05% | eucalyptus oil | This study |
| 2023 | | | Inseticida SBP Aero Multi Leve 450 Pague 300ml | transfluthrin 0.02%, imiprothrin 0.02%, cypermethrin 0.05% | | This study |

| | | | | | | |
|---|---|---|---|---|---|---|
| 2023 | | STRAIK | Straik-Multi Inseticida Aerosol | prallethrin 0.20%, permethrin 0.21% | | This study |
| 2023 | Chile | Baygon | Aygon® Mata Moscas Y Mosquitos | d-fenotrina 0.12 %, d-tetrametrina 0.11%, praletrina 0.05 % | | This study |
| 2023 | | | Baygon® Todo Insecto | d-aletrina 0.1%, tetrametrina 0.3%, d-fenotrina 0.1% | | This study |
| 2023 | | | Baygon® Casa Y Jardín | d-fenotrina 0.1254%, praletrina 0.1003% | | This study |
| 2023 | Colombia | RAID | Raid® Multi | d-fenotrina 0.1254%, praletrina 0.1003% | | This study |
| 2015 | Ecuador | Sapolio | | d-tetramethrin 0.15% | | [9] |
| 2015 | | Raid | | d-tetramethrin 0.35% | | [9] |
| 2015 | | PIX | | chlorpyrifos 0.5% | | [9] |
| 2015 | | Rodasol | | bioallethrin 0.2% | | [9] |
| 2023 | | RAID | Raid® Acción Total | imiprotina 0.031%, cipermetrina 0.100%, praletrina 0.030% | | This study |
| 2023 | | | Raid® Doble Acción Mosquitos Y Moscas | d-fenotrina 0.09784%, d-tetrametrina 0.10369%, praletrina 0.02760% | | This study |
| 2023 | France | RAID | Raid Aerosol Multi-Insects | phenothrin 0.125%, prallethrin 0.1% | | This study |
| 2023 | | | Aerosol Raid Flies & Mosquitoes 2 In 1 | phenothrin 0.125%, prallethrin 0.1% | | This study |
| 2023 | | | Aerosol Raid Flies & Mosquitoes Eucalyptus 2 In 1 | phenothrin 0.125%, prallethrin 0.1% | eucalyptus oil | This study |
| 2003 | Indian | Baygon | | allethrin 0.55% | | [46] |
| 2003 | | Hit | | d-trans allethrin 0.25% | | [46] |
| 2003 | | Mortein | | deltamethrin 0.02%, allethrin 0.13% | | [46] |
| 2023 | Indonesia | Baygon | Baygon Aerosol Citrus Fresh | transflutrin 0,1%, praletrin 0,1%, cypermetrin 0,1% | | This study |
| 2023 | | | Baygon Aerosol Citrus Fresh | transflutrin 0.1%, praletrin 0.1%, sipermetrin 0.1% | | This study |
| 2023 | | | Baygon Aerosol Waterbase | tertrametrin 0.35%, d-fenotrin 0.125%, d-aletrin0.1% | | This study |
| 2023 | | | Baygon Aerosol Natural Orange | d-fenotrin 0,125%, praletrin 0,1% | fragrance of citrus fruits | This study |
| 2023 | | | Baygon Aerosol Tea Blossom | transflutrin 0,1%, praletrin 0,1%, sipermetrin 0,1% | | This study |
| 2023 | | | Baygon Aerosol Eucalyptus | d-fenotrin 0,125%, praletrin 0,1% | eucalyptus oil | This study |
| 2023 | | | Baygon Aerosol Lavender | sipermetrin 0,1%, imiprothrin 0,031%, praletrin 0,03% | Lavender | This study |

| | | | | | | |
|---|---|---|---|---|---|---|
| 2020 | Korea | SC Johnson Korea | F-Killer Max | d-phenothrin 0.98, tetramethrin 1.06, prallethrin 0.28 | | [47] |
| 2020 | | Yuhan Corporation | Happy Home | d-phenothrin 1.35, tetramethrin 2.43 | | [47] |
| 2020 | | Boryung Pharmaceutica l | Home Life Plus | d-phenothrin 0.98, tetramethrin 1.06, prallethrin 0.28 | | [47] |
| 2020 | | Ilmyeong Pharmaceutica l | Green Safe Killer | d-phenothrin 1.10, tetramethrin 1.97 | | [47] |
| 2020 | | SC Johnson Korea | F-Killer Q Water-Based | d-phenothrin 1.20, tetramethrin 1.10, prallethrin 0.50 | | [47] |
| 2020 | | Yuhan Corporation | Happy Home Water-Based | d-phenothrin 1.35, tetramethrin 2.43 | | [47] |
| 2020 | | Henkel Home Care Korea | Home Keeper Water-Based Alpha | d-phenothrin 1.35, tetramethrin 2.43 | | [47] |
| 2015 | Mexico | Killer | | tetramethrin, allethrin, phenothrin | | [38] |
| 2015 | | Raid | | cypermethrin, imiprothrin | | [38] |
| 2015 | | Baygon | | cyfuthrin, imiprothrin | | [38] |
| 2015 | | H24 | | cyfuthrin | | [38] |
| 2015 | | Ortho | | tetramethrin, phenothrin | | [38] |
| 2015 | | Ortho | | permethrin, proxopur, prallethrin | | [38] |
| 2020 | | H24 | H24 Poder Fulminante Ultra Eficaz | propoxur, tetrametrina, fenvalerato | | [48] |
| 2020 | | | H24 Matacucarachas Accion Prolongada ´ Contra Insectos Rastreros | propoxu, prallethrin | | [48] |
| 2020 | | | H24 Domestico Acci ´ On Inmediata | tetramethrin, cifenothrin | | [48] |
| 2020 | | | H24 Poder Citronox | tetramethrin, cifenothrin | | [48] |
| 2020 | | RAID | Raid Max | cypermethrin, imiprothrin | | [48] |
| 2020 | | | Raid Accion Total Mata Cucarachas, Moscas Y Mosquitos | phenothrin, prallethrin, tetramethrin | | [48] |
| 2020 | | | Raid Matamoscas Y Mosquitos Aeroso | phenothrin, prallethrin, tetramethrin | | [48] |
| 2020 | | Baygon | Baygon Ultra Verde | cypermethrin, imiprothrin | | [48] |
| 2020 | | | Baygon Total Insectos Voladores Y Rastreros Uso Domesticot | cyfluthrin, imiprothrin | | [48] |
| 2020 | | Oko | Oko | tetramethrin, cifenothrin | | [48] |
| 2018 | | Baygon | Baygon Casa Y Jardin | prallethrin, phenothrin | | [49] |

| | | | | | | |
|---|---|---|---|---|---|---|
| 2018 | | | Baygon Total | cyfluthrin, imiprothrin | | [49] |
| 2018 | | H24 | H24 Casa Y Jardin | tetramethrin, cifenothrin | | [49] |
| 2018 | | | H24 Citronox | tetramethrin, cifenothrin | | [49] |
| 2018 | | | H24 Mata Moscos Y Mosquitos | tetramethrin, cifenothrin | | [49] |
| 2018 | | Oko | Oko Azul | tetramethrin, cifenothrin | | [49] |
| 2018 | | | Oko Casa Y Jardin | tetramethrin, phenothrin | | [49] |
| 2018 | | | Oko Green Multiusos | tetramethrin, cifenothrin | | [49] |
| 2018 | | | Oko Morado | allethrin, | | [49] |
| 2018 | | Ortho | Ortho Home Defense | tetramethrin, phenothrin | | [49] |
| 2018 | | RAID | Raid Accion Total | imiprothrin, cypermethrin, prallethrin | | [49] |
| 2018 | | | Raid Casa Y Jardin | prallethrin, phenothrin | | [49] |
| 2018 | | | Raid Mata Moscos Y Mosquitos | phenothrin, prallethrin, tetramethrin | | [49] |
| 2023 | | Baygon | Baygon, House and Garden | tetramethrin 0.35%, permethrin 0.10% and allethrin 0.10% | | [50] |
| 2023 | | H24 | H24 Citro Nox | tetramethrin 0.299%, cyphenothrin 0.105 | | [50] |
| 2023 | | | H24 Long-Acting, Ultra-Efficient Fulmi, Mant Power | propoxur 0.46%, tetramethrin 0.103%, fenvalerate 0.455% | | [50] |
| 2023 | | | H 24 | propoxur 0.151%, pra-llethrin 0.0093%, deltamethrin 0.0315% | | [50] |
| 2023 | | RAID | Raid Max With Orange Essence | cypermethrin 0.1%, imiprothrin 0.060% | Orange essence | This study |
| 2023 | | | Raid House And Garden | praletrine 0.100%, phenothrin 0.125% | | This study |
| 2023 | | | Raid House And Garden With Orange Essence | praletrine 0.100%, phenothrin 0.125% | | This study |
| 2023 | | | Raid Kills Flies And Mosquitoes Aerosol | phenothrin 0.12%, pralethrin 0.055, tetramethrin 0.11% | | This study |
| 2023 | | | Raid House And Garden With Eucalyptus Essence | praletrine 0.100%, phenothrin 0.125% | eucalyptus | This study |
| 2023 | Multi countries | RAID | Raid Flying Insect Killer 7 | d-phenothrin (0.125%), prallethrin (0.100%) | | This study |
| 2023 | | | Raid Multi Insect Killer | d-phenothrin (0.125%), prallethrin (0.100%) | | This study |
| 2023 | | | Raid House & Garden I | d-phenothrin (0.125%), prallethrin (0.100%) | | This study |
| 2023 | | Mortein | Powergard Crawling Insect Killer | imiprothrin 0.02%, bioallethrin 0.12%, permethrin 0.05% | | This study |

| | | | | | | |
|---|---|---|---|---|---|---|
| 2023 | | | Mortein Powergard Easy Reach Surface Spray Citrus 350g | cypermethrin 0.2%, imiprothrin 0.01% | | This study |
| 2023 | | | Powergard Insect Spray Multi Insect Killer 300g | imiprothrin 0.07%, permethrin 0.03%, esbiothrin 0.1% | | This study |
| 2023 | | | Mortein Naturgard Fly & Mosquito Killer Eucalyptus Scent | pyrethrins 1%, piperonyl butoxide (pbo) 2% | eucalyptus essence / PBO | This study |
| 2023 | | | Fast Knock Down Multi Inect Killer | imiprothrin 0.05%, permethrin 0.05%, esbiothrin 0.1% | | This study |
| 2023 | | | Mortein Naturgard Multi Insect Killer | pyrethrins 1%, piperonyl butoxide (PBO) 2% | PBO | This study |
| 2023 | | | Mortein Fast Knockdown Crawling Insect Killer Odourless | permethrin 0.3% | | This study |
| 2023 | Nigeria | RAID | Raid Multi-Purpose Insect Killer | d-allethrin 0.250%, tetramethrin 0.15%, deltamethrin 0.015% | | This study |
| 2023 | | | Raid Flying Insect Killer | sumithrin 0.125%, prallethrin 0.100% | | This study |
| 2023 | | | Raid Multi-Purpose Insect Killer | d-allethrin 0.250%, tetramethrin 0.15%, deltamethrin 0.015% | | This study |
| 2023 | Peru | RAID | Raid Full Action | imiprotina 0.031%, cypermethrin 0.100%, pralethrim 0.030% | | This study |
| 2023 | | | Raid Multi | d-phenothrin 01254%, pralethrin 0.1003% | | This study |
| 2023 | | | Raid Mosquitoes And Flies | praletrina 0,02760%, d-tetrametrina 0.10369%, d-fenotrina 0.09784% | | This study |
| 2023 | | | Raid Double Action Mosquitoes And Flies | d-fenotrina 0.09784%, d-tetrametrina 0.10369%, praletrina 0.02760% | | This study |
| 2023 | Philippines | Baygon | Baygon Protector Multi Insect Killer | cypermethrin, imiprothrin, prallethrin | | This study |
| 2023 | | | Baygon Multi-Insect Killer | imiprothrin, cyfluthrin | | This study |
| 2023 | | | Baygon Multi-Insect Killer - Waterbased | tetramethrin, d-phenothrin, d-allethrin | | This study |
| 2023 | | | Baygon Protector Flying Insect Killer | tetramethrin, d-phenothrin, d-allethrin | | This study |
| 2023 | | | Baygon Multi Insect Killer Odorless | imiprothrin, cyfluthrin,prallethrin | | This study |
| 2023 | Poland | RAID | Raid Against Flying Insects With The Smell Of Orange Blossom Aerosol | phenotrin 0.0125%, pralethrin 0.010% | Orange essence | This study |
| 2023 | | | Raid Universal For Running And Aerosol Flying Insects | phenotrin 0.0125%, pralethrin 0.010% | | This study |
| 2023 | | | Raid Against Flying Insects With Eucalyptus Oil Spray | phenotrin 0.0125%, pralethrin 0.010% | eucalyptus oil | This study |
| 2023 | Portugal | RAID | Raid Super Rápido Moscas E Mosquitos Protetor Interiores | piretrina 0,25%; butóxido de piperonilo 1,05% | PBO | This study |

| | | | | | | |
|---|---|---|---|---|---|---|
| 2023 | | | Raid Casa E Plantas | phenotrin 0.0125%, pralethrin 0.010% | | This study |
| 2023 | | | Raid  Multi-Insectos | phenotrin 0.0125%, pralethrin 0.010% | | This study |
| 2023 | South Africa | RAID | Raid Multi Insect Killer | imiprothrin 0.034%, prallethrin 0,04%, tetramethrin 0,2%, piperonyl butoxide (PBO) 1.15% | PBO | This study |
| 2023 | | | Raid Multi Insect Killer Lavender | imiprothrin 0.034%, prallethrin 0,04%, tetramethrin 0,2% | Lavender essence | This study |
| 2021 | Thailand | named -AS1 | | d-phenothrin 0.125%, prallethrin 0.03% | | [51] |
| 2021 | | named -AS2 | | cypermethrin 0.10%, imiprothrin 0.03%, prallethrin 0.03% | | [51] |
| 2021 | | named -AS3 | | pyrethrum 0.1%, piperonyl butoxide (PBO) 0.4% | PBO | [51] |
| 2021 | | named -AS4 | | transfluthrin 0.06%, cyfluthrin 0.03% | | [51] |
| 2021 | | named -AS5 | | permethrin 0.07%, d-tetramethrin 0.14% | | [51] |
| 2023 | | Baygon | Baygon Spray Gets Rid Of Mosquitoes Ants, Cockroaches, Green Recipes | cypermethrin 0.1%, prallethrin 0.03%, imiprothrin 0.03% | | This study |
| 2023 | Turkey | RAID | Raid Flying Insect Killer 7 | d-phenothrin 0.125%, prallethrin 0.100% | | This study |
| 2023 | | | Raid Multi Insect Killer | d-phenothrin 0.125%, prallethrin 0.100% | | This study |
| 2023 | | | Raid House & Garden I | d-phenothrin 0.125%, prallethrin 0.100% | | This study |
| 2023 | UK | Rentokil | Rentokil Rklps136 Insectrol Insect Killer | permethrin 0.46%, d-allethrin  0.13% | | This study |
| 2023 | | Zero | Zero In Fly & Wasp Killer | tetramethrin, permethrin, piperonyl butoxide (PBO) | PBO | This study |
| 2023 | | RAID | Raid Rapid Action Fly Wasp Killer Spray Flying Insect Insecticide | phenothrin 0.05%, prallethrin 0.05% | | This study |
| 2023 | USA | RAID | Raid Flying Insect Killer 7 | d-phenothrin 0.125%, prallethrin 0.100% | | This study |
| 2023 | | | Raid Multi Insect Killer | d-phenothrin 0.125%, prallethrin 0.100% | | This study |
| 2023 | | | Raid House & Garden I | d-phenothrin 0.125%, prallethrin 0.100% | | This study |
| 2023 | Vietnam | RAID | Raid Insect Aerosol - Citrus Flavor | cypermethrin  0.10%, prallethrin 0.03%, imiprothrin 0.03% | citrus essence | This study |

| | | | | | | |
|---|---|---|---|---|---|---|
| 2023 | | | Raid Insect Aerosol - Lavender Incense | cypermethrin 0.10%, prallethrin 0.03%, imiprothrin 0.03% | Lavender essence | This study |
| 2023 | | | Insecticidal Aerosol - Eucalyptus Oil Incense | prallethrin 0.028%, d-phenothrin 0.098%, d-tetramethrin 0.106% | eucalyptus essence | This study |
| 2023 | | | Raid Insect Aerosol - Odorless | cypermethrin 0.10%, prallethrin 0.03%, imiprothrin o.03% | | This study |
| 2023 | | | Mosquito Aerosol - Citrus Flavor | prallethrin 0.028%, d-phenothrin 0.098%, d-tetramethrin 0.106% | citrus essence | This study |
| 2023 | | | Raid Mosquito Aerosol - Lavender Incense | prallethrin 0.028%, d-phenothrin 0.098%, d-tetramethrin 0.106% | Lavender essence | This study |

**Table S3.** Mortality of four mosquito species exposed to household insecticide formulations

| Species | Locality | Mortality % | Age (day-old) | Protocol / Method | Product | Active ingredients | concentration / discharge | Exposure duration | Reference |
|---|---|---|---|---|---|---|---|---|---|
| *Aedes aegypti* | lab strain /Not available | 85 | 2-5 | Cabin / cylindrical cage | PA1 | prallethrin 0.076%, d-phenothrin 0.046% | 14.19 g (8 sec) | 2 hours | [52] |
| | lab strain /Not available | 80 | 2-5 | Cabin / cylindrical cage | PA2 | prallethrin 0.076%, d-phenothrin 0.046% | 7.58 g (4 sec) | 2 hours | [52] |
| | lab strain /Not available | 100 | 2-5 | Cabin / cylindrical cage | PA3 | transfluthrin 0.040%, cyfluthrin 0.025% | 10.23 g (6 sec) | 2 hours | [52] |
| | Morelos / Mexico | 100 | 2-5 | semi-field / cylindrical cage | H24 Casa y Jardin | Tetramethrin = 0.0478 g/kg, cifenothrin = 0.016 g/kg | 16 (g/kg/room) | 1 hour | [49] |
| | Morelos / Mexico | 100 | 2-5 | semi-field / cylindrical cage | H24 Mata Moscas y Mosquitos | Tetramethrin = 0.042 g/kg, cifenothrin = 0.014 g/kg | 14.35 (g/kg/room) | 1 hour | [49] |
| | Morelos / Mexico | 100 | 2-5 | semi-field / cylindrical cage | Oko Green Multiusos | Tetramethrin = 0.0527 g/kg, cifenothrin = 0.0316 g/kg | 21.1 (g/kg/room) | 1 hour | [49] |
| | Morelos / Mexico | 98.33 | 2-5 | semi-field / cylindrical cage | Oko Morado | Allethrin = 0.0477 g/kg | 14.35 (g/kg/room) | 1 hour | [49] |
| | Morelos / Mexico | 95 | 2-5 | semi-field / cylindrical cage | Baygon Total | Cyfluthrin = 0.0029 g/kg, imiprothrin = 0.0099 g/kg | Not available | 1 hour | [49] |
| | Morelos / Mexico | 94.17 | 2-5 | semi-field / cylindrical cage | Raid Mata Moscas y Mosquitos | Phenothrin= 0.002694 g/kg, prallethrin= 0.011225 g/kg, tetramethrin = 0.024695 g/kg | 22.45 (g/kg/room) | 1 hour | [49] |
| | Morelos / Mexico | 91.67 | 2-5 | semi-field / cylindrical cage | Raid Accion Total | Imiprothrin = 0.0081 g/kg, cypermethrin = 0.0261 g/kg, prallethrin = 0.0078 g/kg | 26.13 (g/kg/room) | 1 hour | [49] |
| | Morelos / Mexico | 91.67 | 2-5 | semi-field / cylindrical cage | Ortho Home Defense | Tetramethrin = 0.02 g/kg, phenothrin = 0.02 g/kg | 10.0 (g/kg/room) | 1 hour | [49] |
| | Morelos / Mexico | 89.17 | 2-5 | semi-field / cylindrical cage | H24 Citronox | Tetramethrin = 0.025 g/kg, cifenothrin = 0.0085 g/kg | Not available | 1 hour | [49] |
| | Morelos / Mexico | 84.17 | 2-5 | semi-field / cylindrical cage | Baygon Casa y Jardin | Prallethrin = 0.0160 g/kg, phenothrin= 0.020 g/kg | 16.05 (g/kg/room) | 1 hour | [49] |
| | Morelos / Mexico | 73.33 | 2-5 | semi-field / cylindrical cage | Raid Casa y Jardin | Prallethrin= 0.0313 g/kg, phenothrin= 0.0391 g/kg | 31.3 (g/kg/room) | 1 hour | [49] |
| | Morelos / Mexico | 71.667 | 2-5 | semi-field / cylindrical cage | Oko Casa y Jardin | Tetramethrin = 0.0258 g/kg, phenothrin = 0.0258 g/kg | 8.15 (g/kg/room) | 1 hour | [49] |
| | Morelos / Mexico | 41.67 | 2-5 | semi-field / cylindrical cage | Oko Azul | Tetramethrin = 0.0488 g/kg, cifenothrin = 0.0293 g/kg | 19.55 (g/kg/room) | 1 hour | [49] |
| | Acim / Mexico | 63 | 2-5 | semi-field / cylindrical cage | Raid House and Garden | tetramethrin, allethrin, phenothrin | 10 s | 20 min | [38] |
| | Itzincab / Mexico | 44 | 2-5 | semi-field / cylindrical cage | Raid House and Garden | tetramethrin, allethrin, phenothrin | 10 s | 20 min | [38] |
| | San Lorenzo / Mexico | 43 | 2-5 | semi-field / cylindrical cage | Raid House and Garden | tetramethrin, allethrin, phenothrin | 10 s | 20 min | [38] |

| | | | | | | | | | |
|---|---|---|---|---|---|---|---|---|---|
| | New Orleans / USA / lab strain | 100 | 2-5 | semi-field / cylindrical cage | Raid House and Garden | tetramethrin, allethrin, phenothrin | 10 s | 20 min | [38] |
| | Chiang Mai Province / Thailand | 88.88 | 1-3 | semi-field / cylindrical cage | AS1 | d-phenothrin 0.125%, prallethrin 0.03% | 2.3 g ( 15 s) | 1 hour | [51] |
| | Chiang Mai Province / Thailand | 100 | 1-3 | semi-field / cylindrical cage | AS2 | cypermethrin 0.1%, imiprothrin 0.03%, Prallethrin 0.028% | 2.3 g ( 15 s) | 1 hour | [51] |
| | Chiang Mai Province / Thailand | 84.44 | 1-3 | semi-field / cylindrical cage | AS3 | Pyrethrum 0.1%, Piperonyl Butoxide (PBO) 0.4% | 2.3 g ( 15 s) | 1 hour | [51] |
| | Chiang Mai Province / Thailand | 100 | 1-3 | semi-field / cylindrical cage | AS4 | Transfluthrin 0.06%, Cyfluthrin 0.03% | 2.0 g ( 15 s) | 1 hour | [51] |
| | Chiang Mai Province / Thailand | 75.55 | 1-3 | semi-field / cylindrical cage | AS5 | Permethrin 0.068%, d-tetramethrin 0.135% | 5.3 g ( 15 s) | 1 hour | [51] |
| | New Orleans / USA / lab strain | 100 | 1-3 | semi-field / cylindrical cage | Baygon, house and garden | Tetramethrin (0.35%), permethrin (0.10%) and allethrin (0.10%) | 0.098 g (8 sec) | 30 min | [50] |
| | New Orleans / USA / lab strain | 100 | 1-3 | semi-field / cylindrical cage | H24 Citro Nox | Tetramethrin (0.299%) and cyphenothrin (0.10%) | 0.011 g (1.5 sec) | 30 min | [50] |
| | New Orleans / USA / lab strain | 100 | 1-3 | semi-field / cylindrical cage | H24 long-acting, ultra-efficient fulmi nant power | Propoxur (0.46%), tetramethrin (0.103%) and fenvalerate (0.455%) | 0.033 g (1.5 sec) | 30 min | [50] |
| | New Orleans / USA / lab strain | 100 | 1-3 | semi-field / cylindrical cage | H24 | Propoxur (0.151%), pra llethrin (0.0093%) and deltamethrin (0.0315%) | 0.005 g (1 sec) | 30 min | [50] |
| | Acapulco / Mexico | 77.08 | 1-3 | semi-field / cylindrical cage | Baygon, house and garden | Tetramethrin (0.35%), permethrin (0.10%) and allethrin (0.10%) | 0.441 g (36 sec) | 30 min | [50] |
| | Acapulco / Mexico | 99.59 | 1-3 | semi-field / cylindrical cage | H24 Citro Nox | Tetramethrin (0.299%) and cyphenothrin (0.10%) | 0.085 g (12 sec) | 30 min | [50] |
| | Acapulco / Mexico | 99.17 | 1-3 | semi-field / cylindrical cage | H24 long-acting, ultra-efficient fulmi nant power | Propoxur (0.46%), tetramethrin (0.103%) and fenvalerate (0.455%) | 0.201 g (9 sec) | 30 min | [50] |
| | Acapulco / Mexico | 97.92 | 1-3 | semi-field / cylindrical cage | H24 | Propoxur (0.151%), pra llethrin (0.0093%) and deltamethrin (0.0315%) | 0.045 g (10 sec) | 30 min | [50] |
| | New Orleans / USA / lab strain | 100 | 2-5 | Peet-Grady / cubical cage | (A) | 1R-trans phenothrin 0.10–0.50%, prallethrin 0.10–0.50% | 5-9 g (3–6 s) | 1 hour | [53] |

| | | | | | | | | | |
|---|---|---|---|---|---|---|---|---|---|
| | New Orleans / USA / lab strain | 100 | 2-5 | Peet-Grady / cubical cage | (B) | imiprothrin 0.040%, permethrin 0.056%, D-trans allethrin 0.108% | 5-9 g (3–6 s) | 1 hour | [53] |
| | Cayman Islands / lab strain | 56.17 | 3-5 | Peet-Grady / cubical cage | (A) | 1R-trans phenothrin 0.10–0.50%, prallethrin 0.10–0.50% | 5-9 g (3–6 s) | 1 hour | [53] |
| | Cayman / Cayman Islands / lab strain | 67.96 | 3-5 | Peet-Grady / cubical cage | (B) | imiprothrin 0.040%, permethrin 0.056%, D-trans allethrin 0.108% | 5-9 g (3–6 s) | 1 hour | [53] |
| *Aedes albopictus* | lab strain / Not available | 100 | 2-5 | Peet-Grady / cylindrical cage | F-killer Max | d-Phenothrin (0.98), tetramethrin (1.06), prallethrin (0.28) | 2.8 g (1 s) | 10 min | [47] |
| | lab strain / Not available | 98.3 | 2-5 | Peet-Grady / cylindrical cage | Happy Home | d-Phenothrin (1.35), tetramethrin (2.43) | 2.3 g (1 s) | 10 min | [47] |
| | lab strain / Not available | 91.7 | 2-5 | Peet-Grady / cylindrical cage | Home Life Plus | d-Phenothrin (0.98), tetramethrin (1.06), prallethrin (0.28) | 1.5 g (1 s) | 10 min | [47] |
| | lab strain / Not available | 100 | 2-5 | Peet-Grady / cylindrical cage | Green Safe Killer | d-Phenothrin (1.10), tetramethrin (1.97) | 1.8 g (1 s) | 10 min | [47] |
| | lab strain / Not available | 40 | 2-5 | Peet-Grady / cylindrical cage | F-killer Q Water-based | d-Phenothrin (1.20), tetramethrin (1.10), | 1.6 g (1 s) | 10 min | [47] |
| | lab strain / Not available | 35 | 2-5 | Peet-Grady / cylindrical cage | Happy Home Water-based | d-Phenothrin (1.35), tetramethrin (2.43) | 1.4 g (1 s) | 10 min | [47] |
| | lab strain / Not available | 40 | 2-5 | Peet-Grady / cylindrical cage | Home Keeper Water-based Alpha | d-Phenothrin (1.35), tetramethrin (2.43) | 1.3 g (1 s) | 10 min | [47] |
| *Anopheles gambiae* | Kisumu / Kenyan / lab strain | 100 | 3-5 | Peet-Grady / cubical cage | (A) | 1R-trans phenothrin 0.10–0.50%, prallethrin 0.10–0.50% | 5-9 g (3–6 s) | 1 hour | [53] |
| | Kisumu / Kenyan / lab strain | 100 | 3-5 | Peet-Grady / cubical cage | (B) | imiprothrin 0.040%, permethrin 0.056%, D-trans allethrin 0.108% | 5-9 g (3–6 s) | 1 hour | [53] |
| | Tiassale / Ivory Coast / lab strain | 68.88 | 3-5 | Peet-Grady / cubical cage | (A) | 1R-trans phenothrin 0.10–0.50%, prallethrin 0.10–0.50% | 5-9 g (3–6 s) | 1 hour | [53] |
| | Tiassale / Ivory Coast / lab strain | 77.75 | 3-5 | Peet-Grady / cubical cage | (B) | imiprothrin 0.040%, permethrin 0.056%, D-trans allethrin 0.108% | 5-9 g (3–6 s) | 1 hour | [53] |
| *Culex quinquefasciatus* | Chiang Mai Province / Thailand | 80.48 | 1-3 | semi-field / cylindrical cage | AS1 | d-phenothrin 0.125%, prallethrin 0.03% | 2.3 g (15 s) | 1 hour | [51] |
| | Chiang Mai Province / Thailand | 95.12 | 1-3 | semi-field / cylindrical cage | AS2 | Cypermethrin 0.1%, Imiprothrin 0.03%, Prallethrin 0.028% | 2.3 g (15 s) | 1 hour | [51] |

| | | | | | | | | | |
|---|---|---|---|---|---|---|---|---|---|
| | Chiang Mai Province / Thailand | 92.68 | 1-3 | semi-field / cylindrical cage | AS3 | Pyrethrum 0.1%, Piperonyl Butoxide (PBO) 0.4% | 2.3 g (15 s) | 1 hour | [51] |
| | Chiang Mai Province / Thailand | 95.12 | 1-3 | semi-field / cylindrical cage | AS4 | Transfluthrin 0.06%, Cyfluthrin 0.03% | 2.0 g (15 s) | 1 hour | [51] |
| | Chiang Mai Province / Thailand | 87.80 | 1-3 | semi-field / cylindrical cage | AS5 | Permethrin 0.068%, d-tetramethrin 0.135% | 5.3 g (15 s) | 1 hour | [51] |

## References


1. Potter, A., A. Jardine, and P.J. Neville, *A survey of knowledge, attitudes, and practices in relation to mosquitoes and mosquito-borne disease in Western Australia.* Frontiers in public health, 2016. **4**: p. 32.
2. Castro, J.S.M., et al., *Desigualdades sociais nas práticas de uso de inseticidas domésticos no município de Niterói-RJ.* Rev. bras. ciênc. saúde, 2019: p. 263-272.
3. Oliveira, L.B.d., et al., *Perfil do uso populacional de inseticidas domésticos no combate a mosquitos.* 2015.
4. Galdiano, L.L.d.S., et al., *Household pesticide exposure: an online survey and shelf research in the Metropolitan Region of Rio de Janeiro, Brazil.* Cadernos de Saúde Pública, 2021. **37**: p. e00099420.
5. Hayd, R.L.N. and L.Q. da Silva, *Presença de resistência em Aedes aegypti associada ao uso de inseticidas domésticos em Roraima.*
6. Chan, E.Y.Y., et al., *Sociodemographic predictors of knowledge, mosquito bite patterns and protective behaviors concerning vector borne disease: The case of dengue fever in Chinese subtropical city, Hong Kong.* PLOS Neglected Tropical Diseases, 2021. **15**(1): p. e0008993.
7. N'Dri, B.P., et al., *Use of insecticides in agriculture and the prevention of vector-borne diseases: population knowledge, attitudes, practices and beliefs in Elibou, South Côte d'Ivoire.* Tropical medicine and infectious disease, 2020. **5**(1): p. 36.
8. Stewart Ibarra, A.M., et al., *A social-ecological analysis of community perceptions of dengue fever and Aedes aegypti in Machala, Ecuador.* BMC public health, 2014. **14**(1): p. 1-12.
9. Heydari, N., et al., *Household dengue prevention interventions, expenditures, and barriers to Aedes aegypti control in Machala, Ecuador.* International journal of environmental research and public health, 2017. **14**(2): p. 196.
10. Ryan, S.J., et al., *Seasonal and geographic variation in insecticide resistance in Aedes aegypti in southern Ecuador.* 2019. **13**(6): p. e0007448.
11. Fritzell, C., et al., *Knowledge, attitude and practices of vector-borne disease prevention during the emergence of a new arbovirus: implications for the control of chikungunya virus in French Guiana.* PLoS Neglected Tropical Diseases, 2016. **10**(11): p. e0005081.
12. Kudom, A.A., B.A. Mensah, and J. Nunoo, *Assessment of anti mosquito measures in households and resistance status of Culex species in urban areas in southern Ghana: Implications for the sustainability of ITN use.* Asian Pacific journal of tropical medicine, 2013. **6**(11): p. 859-864.
13. Okyere, C.Y., *Evaluation of alternative mosquito control measures on malaria in Southern Ghana.* Scientific African, 2021. **13**: p. e00866.
14. Mouchtouri, V.A., et al., *Knowledge, attitudes, and practices about the prevention of mosquito bites and Zika virus disease in pregnant women in Greece.* International journal of environmental research and public health, 2017. **14**(4): p. 367.
15. Aerts, C., et al., *Understanding the role of disease knowledge and risk perception in shaping preventive behavior for selected vector-borne diseases in Guyana.* PLoS neglected tropical diseases, 2020. **14**(4): p. e0008149.

16. Chitra, G.A., et al., *High prevalence of household pesticides and their unsafe use in rural South India.* International journal of occupational medicine and environmental health, 2013. **26**: p. 275-282.
17. Dhawan, G., et al., *Malaria-related knowledge and prevention practices in four neighbourhoods in and around Mumbai, India: a cross-sectional study.* Malaria journal, 2014. **13**(1): p. 1-11.
18. Vala, M.C., et al., *Knowledge and practice regarding Malaria among people of urban and rural areas of Rajkot District, Gujarat, India.* Int J Res Med, 2013. **2**(4): p. 38-42.
19. Mehta, D., et al., *A study on knowledge, attitude & practice regarding mosquito borne diseases in an urban area of Bhavnagar.* Health, 2015. **6**: p. 29-32.
20. Nanjesh Kumar, S., et al., *A study of mosquito borne diseases awareness, attitude and practices among the rural population in Karnataka, India.* Int. J. Com. Med. Pub. Health, 2017. **4**: p. 4178.
21. Gorantla, M., S. Katkuri, and P. Kokiwar, *A cross sectional study on knowledge, attitude and practices regarding vector borne diseases in the urban field practice area of a medical college.* International Journal of Community Medicine and Public Health, 2017. **4**(1).
22. Bhardwaj, R., K.S. Mohandas, and M.M. Mathew, *Knowledge, attitude, and practice regarding mosquito-borne diseases among migrant laborers from a migrant settlement in Ponekkara, Ernakulam Kerala.* Indian Journal of Public Health, 2022. **66**(Suppl 1): p. S56-S59.
23. Singh, R., S. Haq, and R. Dhiman, *Studies on knowledge, attitude and practices in malaria endemic tribal areas of Bihar and Jharkhand, India.* J Trop Dis, 2013. **1**(3): p. 1000110.
24. Van Eijk, A.M., et al., *The use of mosquito repellents at three sites in India with declining malaria transmission: surveys in the community and clinic.* Parasites & vectors, 2016. **9**: p. 1-13.
25. Sayono, S., et al., *Distribution of voltage-gated sodium channel (Nav) alleles among the Aedes aegypti populations in Central Java Province and its association with resistance to pyrethroid insecticides.* PLoS One, 2016. **11**(3): p. e0150577.
26. Versari, A., D.M. Sukendra, and S.A. Wulandhari, *Overview of Domestic and Agricultural Pesticides Use Contributing to Aedes aegypti Resistence in Ambarawa Subdistrict, Indonesia.* Unnes Journal of Public Health, 2021. **10**(1): p. 100-109.
27. Widiastuti, D., T. Isnani, and W.S. Sunaryo, *Effectiveness of household insecticides to reduce Aedes aegypti mosquitoes infestation: a community survey in Yogyakarta, Indonesia.* Indian J Public Heal Res Dev, 2018. **9**(6).
28. Sayono, S., et al., *Effect of D-allethrin aerosol and coil to the mortality of mosquitoes.* Journal of arthropod-borne diseases, 2019. **13**(3): p. 259.
29. Sulistyawati, S., et al., *Dengue vector control through community empowerment: lessons learned from a community-based study in Yogyakarta, Indonesia.* International journal of environmental research and public health, 2019. **16**(6): p. 1013.
30. Alobuia, W.M., et al., *Knowledge, attitude, and practices regarding vector-borne diseases in Western Jamaica.* Annals of global health, 2015. **81**(5): p. 654-663.
31. Al-Dubai, S., et al., *Factors affecting dengue fever knowledge, attitudes and practices among selected urban, semi-urban and rural communities in Malaysia.* Southeast Asian J Trop Med Public Health, 2013. **44**(1): p. 37-49.
32. Md, S.M., A. Er, and J. Pereira, *Identifying the sources of dengue infection and prevention practices at household level in Seremban district, Malaysia.* Journal of Environmental Science and Technology, 2017. **10**(5): p. 238-244.
33. Selvarajoo, S., et al., *Knowledge, attitude and practice on dengue prevention and dengue seroprevalence in a dengue hotspot in Malaysia: A cross-sectional study.* Scientific reports, 2020. **10**(1): p. 9534.
34. Azratul-Hizayu, T., et al., *Knowledge, Attitudes, and Practices on the Use of Household Insecticide Products: Is the Awareness in Place?* Journal of Integrated Pest Management, 2021. **12**(1): p. 48.
35. Wong, L.P., et al., *Factors affecting dengue prevention practices: nationwide survey of the Malaysian public.* PloS one, 2015. **10**(4): p. e0122890.

36. Legorreta-Soberanis, J., et al., *Household costs for personal protection against mosquitoes: secondary outcomes from a randomised controlled trial of dengue prevention in Guerrero state, Mexico.* BMC Public Health, 2017. **17**(1): p. 151-158.
37. Loroño-Pino, M.A., et al., *Household use of insecticide consumer products in a dengue-endemic area in México.* Tropical Medicine & International Health, 2014. **19**(10): p. 1267-1275.
38. Gray, L., et al., *Experimental evaluation of the impact of household aerosolized insecticides on pyrethroid resistant Aedes aegypti.* 2018. **8**(1): p. 1-11.
39. Iftikhar, B., et al., *KNOWLEDGE AND PRACTICE OF GENERAL POPULATION REGARDING VECTOR BORNE DISEASES.* Journal of Medical Sciences, 2016. **24**(3): p. 185-189.
40. Yboa, B.C., et al., *Dengue knowledge and preventive practices among rural residents in Samar province, Philippines.* Int Jou Pub Health Sci, 2013. **2**(2): p. 59-66.
41. Kumanan, T. and D. Logeswaran, *A study on knowledge, attitude and practices regarding dengue among hospitalized patients from Northern Sri Lanka.* 2018.
42. Nalwanga, E. and J.C. Ssempebwa, *Knowledge and practices of in-home pesticide use: a community survey in Uganda.* Journal of environmental and public health, 2011. **2011**.
43. Haenchen, S.D., et al., *Mosquito avoidance practices and knowledge of arboviral diseases in cities with differing recent history of disease.* The American journal of tropical medicine and hygiene, 2016. **95**(4): p. 945.
44. Bohmann, A.K., et al., *Personality and city culture predict attitudes and practices toward mosquitoes and mosquito-borne diseases in South Texas.* Frontiers in Public Health, 2022. **10**: p. 919780.
45. Jumbam, D.T., et al., *Knowledge, attitudes and practices assessment of malaria interventions in rural Zambia.* BMC Public Health, 2020. **20**(1): p. 1-15.
46. Snehalatha, K., et al., *The mosquito problem and type and costs of personal protection measures used in rural and urban communities in Pondicherry region, South India.* Acta tropica, 2003. **88**(1): p. 3-9.
47. Yoon, J., et al., *Efficacy of seven commercial household aerosol insecticides and formulation-dependent toxicity against Asian Tiger Mosquito (Diptera: Culicidae).* Journal of Medical Entomology, 2020. **57**(5): p. 1560-1566.
48. Dzib-Florez, S., et al., *Bio-efficacy of commercially available residual insecticides for the control of Aedes aegypti in Mexico.* Journal of the American Mosquito Control Association, 2020. **36**(1): p. 16-21.
49. Kuri-Morales, P.A., et al., *Efficacy of 13 commercial household aerosol insecticides against Aedes aegypti (Diptera: Culicidae) from Morelos, Mexico.* Journal of Medical Entomology, 2018. **55**(2): p. 417-422.
50. López-Solis, A.D., et al., *Evaluation of commercial aerosol insecticides for control of Aedes aegypti susceptible or resistant to pyrethroids.* salud pública de méxico, 2023. **65**(2 mar-abr): p. 151-159.
51. Nachaiwieng, W., et al., *Efficacy of five commercial household insecticide aerosol sprays against pyrethroid resistant Aedes aegypti and Culex quinquefasciatus mosquitoes in Thailand.* Pesticide Biochemistry and Physiology, 2021. **178**: p. 104911.
52. Khadri, M., et al., *Efficacy of commercial household insecticide aerosol sprays against Aedes aegypit (Linn.) under simulated field conditions.* Southeast Asian journal of tropical medicine and public health, 2009. **40**(6): p. 1226.
53. Silva Martins, W.F., et al., *Improving the efficiency of aerosolized insecticide testing against mosquitoes.* Scientific Reports, 2023. **13**(1): p. 6281.
